# Wild Edible Plants of Andalusia: Traditional Uses and Potential of Eating Wild in a Highly Diverse Region

**DOI:** 10.3390/plants12061218

**Published:** 2023-03-07

**Authors:** Guillermo Benítez, Joaquín Molero-Mesa, M. Reyes González-Tejero

**Affiliations:** Departamento de Botánica, Universidad de Granada, Campus Universitario de Cartuja, 18071 Granada, Spain

**Keywords:** ethnobotany, conservation of plant resources, crop relatives, food plants

## Abstract

A review of ethnobotanical sources focused on traditionally-used wild food plants in Andalusia (southern Spain), one of the most biodiverse regions in Europe, is carried out. With 21 original sources plus some previously unpublished data, the dataset shows a high diversity of these traditional resources, reaching 336 species or c. 7% of the total wild flora. Cultural aspects related to the use of some species are discussed and data are compared with similar works. The results are discussed through the lens of conservation and bromatology. For 24% of the edible plants, informants also mentioned a medicinal use (achieved by consuming the same part of the plant). In addition, a list of 166 potentially edible species is provided based on a review of data from other Spanish territories.

## 1. Introduction

The term ‘wild foods’ (WFs) has been used to describe all plant resources that are harvested or collected for human consumption outside of agricultural areas, e.g., in forests, savannahs, and other shrub-land areas [1]. A wide variety of WFs, including fruits, leafy vegetables, woody foliage, bulbs and tubers, cereals and grains, nuts and kernels, saps and gums (eaten or used to make drinks), mushrooms, terrestrial invertebrates (insects, snails, etc.), honey, birds’ eggs, fish, shellfish, and meat from small and large vertebrates [2] contribute to the diets of large numbers of people [3]. Wild edible plants (WEPs) and, particularly, the consumption of traditional leafy vegetables (wild or leafy greens) as an important source of micronutrients are attracting a great deal of attention. These traditional leafy vegetables represent a valuable resource in several Mediterranean countries, such as France, Greece, Italy, Spain, and Turkey [1]. This is because, among the WFs, the most diverse and most frequently gathered group is that of wild edible plants (WEPs) [3]. Although previously neglected [4], the topic is now receiving renewed attention.

Uniformity in the markets now favours the demand for different crop varieties or species, both by consumers and, consequently, companies in this sector. With this standardization of foods and ingredients, WFs can be an important source of dietary diversity. On the other hand, while a large amount of information has been accumulated on the characteristics of the domesticated species, many gaps in this information remain, particularly for species, varieties, and breeds that are not widely used commercially. Thus, information on WFs is also often limited [3]. Efforts are being made to develop a body of literature on the nutrient composition and medicinal properties of wild foods (e.g., [5,6,7]). Other data (molecular-genetic data, ecogeographical data, vernacular names, parts used, modes of preparation, specific uses, seasonal harvest and use patterns, and traditional knowledge related to various aspects of management) can all be important in planning the sustainable use and conservation of wild food species [3]. Whilst WEPs are regularly deprecated by policy makers and considered to be “weeds of agriculture,” it would be tragic if this were to led to the loss of the ability to identify and consume these important available species [8].

Mediterranean diets are highly diverse and local, and are influenced by the cultural and biological diversity of each territory [9,10,11]. Previous research concluded that out of about 2300 different wild plant species used as food in the Mediterranean, approximately 1000 are only consumed locally in a single area [11]. The use of WEPs requires special cultural knowledge regarding harvesting, preparation, cooking, and other forms of processing, and, in most cases, the species are managed, tended or manipulated in some way to increase their productivity and availability [12]. Furthermore, studies have shown the importance of wild plants as functional foods [13,14,15], providing essential micro- and macronutrients [7,8,16,17]. Many WFs are rich in micronutrients, some containing more than their cultivated counterparts ([18,19] studies for Brazil and Burkina Faso). Eating them can alleviate micronutrient and/or protein deficiencies and therefore make diets more nutritious and balanced [3,20]. Additionally, we must not forget the phytotherapeutic aspects of these species, as many are consumed in search of a double nutritional and therapeutic benefit [21,22]. As Etkin [4] stated, these sources distil the striking nutritional and pharmacological potential of wild plants and their cultural implications.

Moreover, global food security is unfortunately under threat, and one of the main issues is climate change. The processes we have been observing have already affected crop suitability in many areas, resulting in changes in the production of major agricultural crops [23]. In this scenario, WFs can contribute to food security through direct consumption (either regularly or in times of scarcity) and when sold to generate income that could be reinvested in other food purchases [3]. WFs, while forming a significant proportion of the global food basket, are excluded from official statistics on the economic values of natural resources [24]. Thus, WFs can help households to cope with fluctuations in the supply of food or income-generating opportunities [25]. A wide range of such foods are often important components of the diet or sources of income during lean seasons of the year or in times of drought or other disasters, such as food shortages [3]. WFs could therefore be a potential solution to help overcome food insecurity [26], as well as acting as an important genetic reservoir that can help improve their cultivated relatives.

A number of reports from several countries highlight the need for a greater recognition of the contribution that WFs make to global food security and nutrition. Estimations from African and Asian countries have led to a mean of 120 wild species being used as wild foods per community, with aggregated country estimations reaching 300–800 species for countries such as India, Ethiopia, or Kenya [24]. However, it is clear that there are considerable gaps in knowledge with regard to the extent of this contribution in quantitative terms. Several countries note the need to collect more data on wild food use, for example, by including WFs in national censuses and surveys or in ethnobiological or other scientific studies. Limitations in terms of capacity development and stakeholder involvement are also highlighted. Some countries identify a need to increase the knowledge on the effects of wild food use on human health and well-being, including, in some cases, not only the nutritional impacts, but also the effects on cultural life and the possible stress-reducing effects of collecting wild foods [3]. Although WFs may not be particularly diverse in the European, Central Asian, and North American regions, the status and trends of WFs are better monitored in these regions than elsewhere [3]. The habit of gathering plants from the wild has endured for centuries, linked especially to rural societies. These plants provided a source of food for many people in times of war and famine, situations that became frequent during the 16th to 19th centuries [27].

Research data show that the diversity of WEPs is generally underestimated and poorly understood. In the Mediterranean region c. 2300 different plant and fungal taxa are gathered from the wild for consumption [10]. Focusing on Spain, information on WEPs is available for some territories (e.g., [28,29,30,31,32,33,34]), and a national review has listed the considerable amount of 419 species of WEPs [35]. Nevertheless, Andalusia remains understudied.

### Hypothesis and Aim of the Study

Given the high plant biodiversity of the study area, its long history of human settlement and cultural diversity, and the availability of a considerable amount of ethnobotanical literature, the diversity of plant resources used as food in Andalusia is under-known. Our aim with this work is to make it known and to analyse its potential.

The goal of this study was to compile, describe, and assess data on traditionally used WEPs from Andalusia. As a secondary goal, we aimed to offer information regarding the potential use of other species as WEPs which, growing wildly in the territory, are also being used in haute cuisine restaurants that use wild plants in their dishes.

## 2. Results

The list of included species with uses, vernacular names, edible use categories, parts of the plants used, and original sources is presented in Appendix A. We compiled the edible uses of 336 WEPs in Andalusia, belonging to 127 genera and 70 botanical families. A total of 428 edible uses and 2435 total citations have been recorded with the described mixed method (1288 UR from informants and the rest recorded by counting the number of sources mentioning each WEP).

Outstanding genera are: *Thymus*, with eight species (one with two subspecies) used as seasonings; *Prunus*, with seven species (two naturalized and five wild) of edible fruits (e.g., the endemic *Prunus ramburii* Boiss.); *Rumex*, with seven species; and *Allium*, with six species of edible bulbs. Other important genera with five edible wild species each are: *Asparagus* (edible tender shoots), *Lactuca* (edible leaves), *Lonicera* (flowers used as a snack), and *Malva* (with fruits eaten as snacks and, in some species, leaves as green vegetables).

The review was focused on plants growing wild in Andalusia. Nevertheless, we also included 28 species that were considered naturalised or sub-spontaneous (e.g., *Robinia pseudoacacia* L. or *Melissa officinalis* L., Appendix A) according to Blanca et al. [36]. Cultivated Rosaceae with edible fruits can also grow near cultivated lands, but they have not been included as WEPs except for cases in which naturalization has been clearly detected and is clearly mentioned in [36] (e.g., *Prunus domestica* L. and *Prunus dulcis* (Mill.) D.A. Webb). Other cultivated species, e.g., *Malus domestica* (Borkh.) Borkh., *Cydonia oblonga* Mill., *Prunus armeniaca* L., *Prunus cerasus* L., *Prunus persica* (L.) Batsch, and *Pyrus communis* L. were logically excluded from our dataset, as were others like *Diospyros kaki* L. f. and *Punica granatum* L.

**Figure 1:**
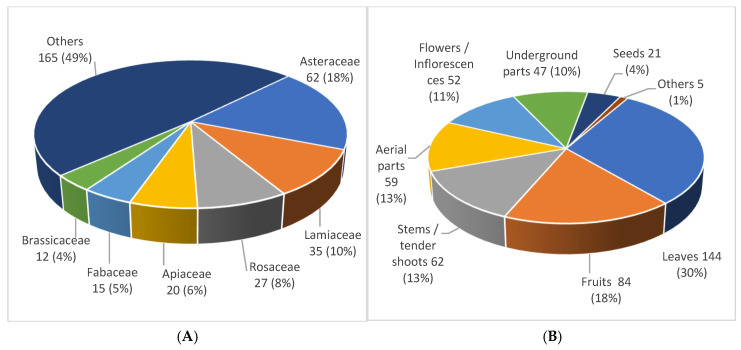
(**A**) Distribution of species among botanical families. (**B**) Distribution of parts of the plant used.

The distribution among botanical families shows that the nine most important families comprise up to 59% of the species. The main families are represented in Figure 1A.

For 40 of the WEPs, this is the first ethnobotanical report documenting their use in Andalusia. Most of them have uses not previously reported in the territory but generally well-known in nearby territories (28 of the plants are cited in the three consulted works). These include, for example, the consumption of the leaves of *Calendula officinalis* L., *Leontodon tuberosus* L., *Cardaria draba* (L.) Desv., and *Diplotaxis erucoides* (L.) DC., as well as the fruits of *Sambucus nigra* L. and *Crataegus laciniata* Ucria. Some are locally well reputed and frequently consumed (e.g., *Rhagadiolus edulis* Gaertn. in rural areas of Granada province).

The distribution of the parts of the plants used (Figure 1B) shows a higher proportion of documented uses involving leaves, which are generally used in salads or soups. It is a heterogeneous group with an abundance of Asteraceae (35 species), Lamiaceae (12), and Brassicaceae (11), as well as the frequent use of Apiaceae and Polygonaceae (9). This is followed by the consumption of fruits, such as those of the Rosaceae species; stems and tender shoots (frequent in genus *Asparagus* and other plants with a similar mode of consumption); and aerial parts (i.e., all parts of the plant above the ground, especially in the case of the use of the Lamiaceae as seasoning). Most of the edible uses were categorised as “food” (55,8%), but “snacks” and “seasonings” (17 and 13% respectively) also stand out.

## 3. Discussion

### 3.1. Andalusian WEPs

As expected, among the WEPs in Andalusia, some species are widely consumed throughout many Mediterranean European countries, e.g., *Sonchus* spp., *Crepis vesicaria* L., *Silene vulgaris* (Moench) Garcke, *Papaver rhoeas* L., *Borago officinalis* L., *Beta maritima* L., *Allium ampeloprasum* L., *Portulaca oleracea* L., *Crataegus monogyna* Jacq., and *Foeniculum vulgare* Mill. (e.g., [11]). In fact, all plants with 20 or more URs (21 species, Table 1) were listed in previous reviews or datasets at both the national and Mediterranean levels [11,35] and are also widely used in other Spanish territories (see Appendix A for references). Additionally, the bromatological and phytochemical profiles of these plants have already been surveyed [7,37].

On the other hand, 135 WEPs have been cited only once and may be underreported or underutilised. Possible reasons for and examples of underreporting these plants are diverse. Some plants have few populations, or populations in protected areas where gathering is forbidden or requires special permission from the government (such as *Allium sphaerocephalon* L. and *Ribes alpinum* L. in the National Park of Sierra Nevada). Plants may also belong to a genus with other species more frequently used as WEPs (*Allium baeticum* Boiss. is only cited in one work, as the species is neither frequent nor abundant, but *A*. *ampeloprasum* L. is among the most cited ones). Furthermore, the local use of endemic species may be restricted to a small distribution area (*Cirsium rosulatum* Talavera and Valdés is only used in Cazorla and surroundings, as it is a local endemism of that territory.). Alternatively, species may be more or less widely present, but only used locally (*Chrysanthemum coronarium* L. was only used as WEP in Cabo de Gata). Meanwhile, plants with few recorded instances as WEPs in the form of a non-medicinal beverage were generally cited as medicinal in the form of herbal teas (*Santolina rosmarinifolia* L. subsp. *canescens* (Lag.) Nyman; *Thymus baeticus* Lacaita). There may also be underreported but well-known edible uses (*Origanum compactum* Benth., widely used in Cadiz but just cited in one work without URs; and *Prunus ramburii* Boiss., gathered for liquors in Granada). Finally, some WEPs may be eaten during times of famine, but while edible may not really taste good or may not provide much nutritional value. Some sources [38,39,40] distinguish between regular WEPs and plants edible only in cases of emergency after the Spanish Civil War (1936–1939). The gathering of WEPs was a means of survival in the years of scarcity after the war, as in other territories and recent conflicts (e.g., [41,42,43]). It has been alluded that, due to the scarcity of food, the collection of wild species increased, which were not always edible [38].

Previous comparative studies within other Mediterranean countries showed that the Spanish traditionally use a higher number of WEPs (51.5% and 15% more than Italy and Greece, respectively) even though only a few species (which are widely used as WEPs) were shared between the three countries [11]. Our data show that nearly 7% of the wild plants of Andalusia have been used as WEPs (considering 4437 plant taxa; [44]). This is similar to the national ratio (7.5%, based on 419 WEPs and a total of 5537 vascular plants in the Iberian Peninsula [22,35,45]). This high amount of local wild resources traditionally used as food can be seen as a culturally positive attitude regarding the use of wild vegetables, which was described as *herbophilia* [46]. In this sense, Andalusia can also be seen as an *herbophilic* territory, in contrast with other types, such us Poland, classified as *aherbous,* or even *herbophobous* [47]. Moreover, only 58% of the included taxa (195 plants, see Appendix A) are recorded as edible in the PFAF database [48], which also compiles other important catalogues (e.g., [49,50]), denoting a high proportion of locally used or under-known plant resources.

It is noteworthy that 33% of the plants included here (121 species) were not previously reported for Spain [35] or in the two recent reviews of territories near Andalusia (i.e., Valencia [51]; and Albacete [31]). Thus, this also represents an important contribution to the knowledge of the species used at a national level.

### 3.2. Bromatology and Medicinal Use

While significant progress has been made in the field of WEP bromatology (e.g., [5,6,7,52]), much research remains to be done in this area. For 35 WEPs (Appendix A), nutritional values and contents are known, and food composition tables can be seen [7]. In this publication, the authors selected 41 plant species extensively consumed in the Mediterranean region (mentioned in four to sixteen different countries). It is noteworthy that, of these 41 species, only four are not cited in Andalusia. Nevertheless, the majority of the Andalusian WEPs (90%) have not been studied in detail, and the nutritional or medicinal benefits that their consumption can provide are unknown, thus representing a challenge for bromatologists, nutritionists, and researchers in the field of food science and technology.

For 83 of the plant species included in the list, informants also listed at least one medicinal use achieved simply by consuming the same part of the plant used as a food. Therefore, 24% of the WEPs can also be considered traditional functional foods, and in our opinion, this merits further study. In general, most of the medicinal uses are related to digestive disorders (70 plants had uses including treating gastralgia, stomach ache, parasites, diarrhoea, constipation, or as appetizers), but others relate to respiratory disorders (32 for cold, cough, bronchitis), circulatory disorders (10), or may be used as diuretics (8), or against kidney (8) or liver (5) disorders, or hypercholesterolemia (5). Some specific medicinal uses of certain species were already noted [7,22,31], but more studies on these folk uses are needed within the scope of ethnopharmacology (outside of the focus of this review).

### 3.3. Conservation of WEPs

Contrary to what is often assumed, evidence demonstrates that a significant proportion of wild food comes from areas used for crop and/or livestock production, or from around the home [53], and Andalusia is no exception. Most of the WF resources are collected in places with anthropogenic vegetation and in grassland and meadow zones, and it seems that people collect these resources mostly from nearby places [54].

The overuse of wild products is a major problem in many places and has implications both for biodiversity and, in the medium term, for the sustainability of the livelihoods of people relying on these resources [3]. Overexploitation is the main threat to the conservation of wild food species [3], followed by habitat alteration and pollution. Nevertheless, in Spain, studies dealing with the most diverse group of wild plants gathered for traditional medicinal practices have demonstrated that this problem is not of great concern (commercial exploitation excluded) since the vast majority of the species used do not experience problems in their conservation and, with few exceptions, are not included in regional or national conservation laws [55]. Thus, the same could be said for WEPs.

Most plant species in Appendix A are not endangered. Gathering from wild populations is only prohibited for six plant species, as they are recorded in the Andalusian list of endangered plants and mushrooms (annex of Decreto 23/2012, Andalusian Government). However, regional regulations concerning the exploitation of wild species on privately owned forest lands (Orden de 2 de junio de 1997, BOJA) also require a specific authorization in order to gather certain species (31 plants mostly Lamiaceae, marked in Appendix A). Lastly, *Ribes alpinum* L. and *R. uva-crispa* L., species frequently found in other Spanish territories but rarely in Andalusia, are vulnerable according to the standards of the IUCN [36].

The role of WEPs in cultures and agroecosystems has been outlined [12]. The simplification of the environmental structure and diversity of the farming activities is clear, as well as the influence of agriculture on the evolution of weedy species and its ecology. However, most WEPs can be considered weeds, and their ecology is to grow close to areas inhabited by humans [22,54]. Their cultivation could be a success from the perspective of the diversification and resilience of agricultural practices. Agroecological farms are particularly suitable for the cultivation of new WEPs to develop novel food products based on low-input systems and the valorization of ecosystem services. Furthermore, some WEPs are more drought-resilient than cultivated plants, permitting water-saving agricultural practices [25].

### 3.4. WEPs Potentially Used (PWEPs)

While the WEPs in Appendix A were recorded through ethnobotanical methods in the original sources and have a cultural background, we are aware that the real potential for wild species in Andalusia to be used as food is much higher. In order to compare the traditional WEPs with the potential of the Andalusian natural landscape, we also present (Supplementary Table S1) a table with potentially exploitable species. In this sense, we define them as potential wild edible plants (PWEPs). In order to be included in this table, the species must have met at least one of the following criteria: 1. they grow wild in Andalusia and can be gathered without cultivation; 2. they have been cited as edible in other Spanish regions (e.g., [28,29,31,35,51,56]); 3. they are currently used in haute-cuisine restaurants in the region (e.g., leaves of *Mesembryanthemum nodiflorum* L. or *Lepidium sativum* L.); and 4. they have been used as food in the past (e.g., stems of *Halogeton sativus* (L.) Moq.).

With these criteria, Supplementary Table S1 presents a tentative list of 166 plant species not included in Appendix A, which may potentially be gathered as food resources. Some of the species have been traditionally consumed in Spain in the past, for example, several halophytes prepared in vinegar similar to *Capparis* fruits (e.g., stems of *Halimione portulacoides* (L.) Aellen or *Halogeton sativus* (L.) Moq.; [57,58]). Some Aizoacae and Chenopodiaceae are currently served in luxury restaurants. Going back into history, in the Spanish-Muslim literature from the Al-Andalus period (7th to 15th centuries) we can find data on the edibility of wild plants that are no longer consumed today, either due to cultural changes or an erosion of the traditional knowledge. We highlight the edible use of *Cynomorium coccineum* L., mentioned by Abu l’Jayr (XI–XII centuries) and Ibn al-Baytar (XIII century) [59], as well as the use of *Rhus coriaria* L. fruits (sumac) as a seasoning, still frequent in the Levant. Even earlier, some wild vegetables in this table were consumed by the Romans, e.g., *Crambe maritima* L. [58]. Finally, we included some species that are relatives of others reported as WEPs on the basis of a probable confusion or indistinct use regarding the species (or even when recognising the genus). As an example, Appendix A lists three species of *Onopordon* whose leaves’ midribs are eaten in times of famine in Granada, Cordoba, and Jaen. In addition, we include two more species as PWEPs for which ethnobotanical data have been reported in other Spanish territories [35].

With this section, we want to draw attention to the local potential of the plant kingdom for human food, even if there is no specific cultural basis that recognises these resources as food resources. This idea of PWEPs has already been used in other territories (e.g., [60]). In our opinion, as long as the cultural contexts of the population residing in the study area and the population interviewed for data collection on plant uses are the same, PWEPs can reflect the real potential for the use of local resources. This does not apply if cultural backgrounds are not similar. Nature is full of examples: while *Solanum nigrum* L. is generally considered toxic in Spain due to its alkaloids and therefore not cited as a WEP (a view also reflected in some vernacular names such as ‘tomatillo del diablo’), its fruit is consumed as food in Bolivia and Peru, and its leaves in Australia, Tanzania, Ethiopia, and Somalia [61].

Adding these PWEPs to the list of WEPs in Appendix A, we achieve a total number of 532 plant species which, growing wild in Andalusia, can be freely gathered for food and reach 12% of the total flora of the region. In a review of the possible ‘solutions for a cultivated planet,’ the authors conclude that “the challenges facing agriculture today are unlike anything we have experienced before, and they require revolutionary approaches to solving food production and sustainability problems. In short, new agricultural systems must deliver more human value, to those who need it most, with the least environmental harm” [62]. In our opinion, WFs can meet some of these challenges, as they are foods produced with little associated environmental damage, and their cultivation and domestication can motivate this revolutionary approach for food production.

## 4. Materials and Methods

### 4.1. Study Area

**Figure 2:**
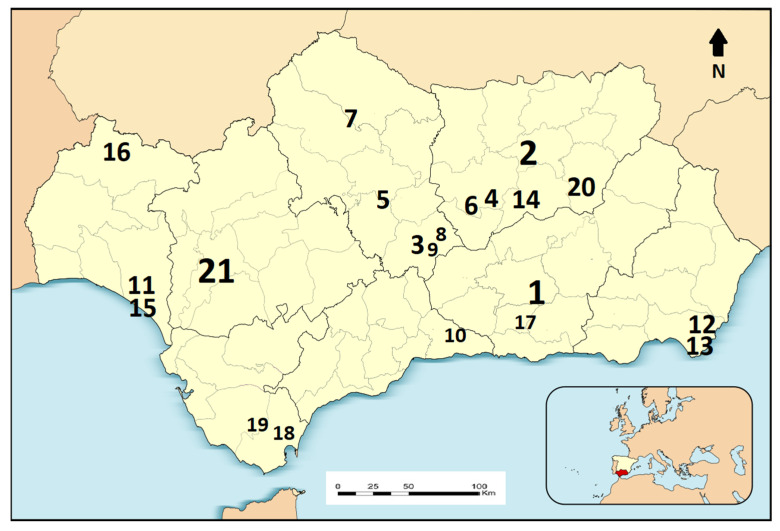
Map of the study area with the locations of the original works. See Table 2 for proper citations and territories covered in each original source. Large numbers are for provincial studies, medium for regional studies, and small for county or municipal studies.

The review covers the whole territory of Andalusia, one of the 17 autonomous communities of Spain (Figure 2). Covering 87,268 km^2^, the region has a highly diverse physical environment, with altitudes from sea level up to 3479 metres a.s.l., several mountain ranges, a high diversity of geology and soils, and a Mediterranean climate with a great variety of microclimates [63]. Andalusia is, together with the Rif, one of the two main centres of biodiversity in the Mediterranean basin (the other includes parts of Turkey and Greece; [44,64]). The checklist of vascular flora includes 4437 plant taxa distributed across 171 botanical families [44]. It is the most diverse region on the Iberian Peninsula in terms of vegetation types [65].

In addition to the species, vegetation, and landscape diversity, cultural and historical factors should also be considered [66]. Andalusia is one of the most populated regions in Europe [67] and has been so since ancient times. Multiple cultures of the Mediterranean have passed through the area, which has also been central to trade with other regions of the world from the 15th century onwards. Consequently, the diversity of plant resources used in traditional ways is high.

### 4.2. Review Method and Inclusion Criteria

The review was focused on ethnobotanical field works which were based on direct data collection with local informants in which traditional knowledge was sought. We first selected opportune ethnobotanical literature from Andalusia, dealing with edible plants (see Table 2 for original sources, types of works, citations, and other interesting data for each). The ethnobotanical literature dealing not with edible resources, but with medicinal ones (e.g., [68,69]) was omitted.

**Table 2:** Original sources for the dataset with citations, territories covered, types of works, and specifying whether use reports (UR) and the part of the plant used in each case were mentioned. UP: Unpublished work. *: UR not specified but with verbatim quotations from the informants from which URs were retrieved.

Ref	Citation	Territory	Kind of Work	UR	Used Part
1	Benítez et al., 2017 [22]	Granada province	Paper (ethnobotany)	Yes	Yes
2	Guzmán Tirado, 1997 [70]	Jaen province	PhD dissertation (ethnobotany), UP	Yes	Yes
3	Triano et al., 1998 [71]	South-eastern Cordoba province	Booklet (ethnobotany)	No	Yes
4	Casado Ponce, 2003 [72]	Southern Jaen province	PhD dissertation (ethnobotany), UP	No *	Yes *
5	Casana Martínez, 1993 [73]	Southern Cordoba province	PhD dissertation (ethnobotany), UP	No	Yes
6	Ortuño Moya, 2004 [74]	Los Villares and Valdepeñas region, Jaen province	PhD dissertation (ethnobotany), UP	No *	Yes *
7	Galán Soldevilla, 1993 [75]	Northern Cordoba province	PhD dissertation (ethnobotany), UP	No *	Yes
8	Molina Mahedero, 2001 [76]	Carcabuey, Cordoba province	Degree dissertation (ethnobotany), UP	No	Yes
9	Sánchez Romero, 2003 [77]	Rute, Cordoba province	Degree dissertation (ethnobotany), UP	No	No
10	López García, 2015 [78]	Frigiliana, Malaga province	Master dissertation (ethnobotany), UP	Yes	Yes
11	Rodríguez Franco, 2013 [79]	Doñana region	Degree dissertation (ethnobotany), UP	Yes	Yes
12	Martínez-Lirola et al., 1996 [80]	Cabo de Gata region, Almeria province	Paper in scientific journal (ethnobotany)	Yes	Yes
13	Martínez-Lirola, 1993 [81]	Cabo de Gata region, Almeria province	Degree dissertation (ethnobotany), UP	Yes	Yes
14	Mesa Jiménez, 1996 [82]	Sierra Magina, Jaen province	PhD dissertation (ethnobotany), UP	No	Yes
15	Cobo and Tijera 2011 [83]	Doñana region	Book (ethnobotany)	No	Yes *
16	Hadjichambis et al., 2008 [84]	Aracena mountains, Huelva (Circunmediterranean diverse territories)	Paper (ethnobotany)	No	Yes
17	Gil Palomo and Juárez Castillo, 2005 [85]	Castaras, Granada province	Booklet (ethnobotany)	No	Yes
18	Velasco et al., 1998 [86]	Campo de Gibraltar region, Cadiz province	Paper (ethnobotany)	No	No
19	Velasco et al., 2000 [87]	Los Alcornocales Natural Park, Cadiz province	Paper (ethnobotany)	No	No
20	Fernández Ocaña, 2000 [39]	Cazorla, Segura y las Villas Natural Park, Jaen province	PhD dissertation (ethnobotany)	No *	No
21	González Turmo, 1995 [38]	Eastern Andalusia (general)	Book	No	Yes

All the original sources followed general ethnobotanical field methods based on semi-structured interviews along with the proper identification of the plant material gathered with the informants using local flora (for more details on field work, see f.i., [22,40] Benítez, 2009, or Benítez et al., 2017). Voucher numbers for each species can be consulted in the original sources. Botanical nomenclature and families were standardized using Blanca et al. [36,44] (2009) and Cueto et al. (2018). As the complete list of the vernacular names of some species is long, we only mention the most used names according to the consulted sources. This paper does not aim to review the traditional medicinal uses of the included species. The included medicinal uses from the consulted literature only apply to the edible parts of each plant and are only achieved when local people specifically eat this edible part [22] (Benítez et al., 2017). Thus, other medicinal uses involving other parts of the plant or modes of application other than ingestion (e.g., external uses) are not included (e.g., when the medicinal use requires a specific preparation other than eating the edible part or drinking the specific drink generally prepared for food and not as a medicinal beverage, it was not included).

We also report previously unpublished data on the use of some species as food. These are based on our research team’s ethnobotanical records, which were collected using the same methods described above.

### 4.3. Categorization of Edible Uses

Using the specific consumption forms filled out by the informants, we classified edible uses into different categories: (i) foods, when ingested in any way, cooked or raw, such as when fried, boiled, used in salads or omelettes, etc., including wild fruits; (ii) snacks, when ingested only for their special, pleasant taste, such as the extraction of nectar from flowers, small fruits eaten raw without seeking any nutritional property, etc.; (iii) seasonings, when added to any traditional recipe; (iv) liqueurs, when an alcoholic beverage is prepared with a plant; (v) drinks, for plants prepared in infusions or decoctions with water without seeking a medicinal property; and (vi) curds, for plants used to make homemade cheese. In this sense, note that according to this classification, parts of edible plants eaten raw cannot be locally considered a food but a snack, either because it is seen as too small of an amount when ingested, or because the intake is valued more for its flavour than for its nutritional properties [22] (Benítez et al., 2017).

We use a mixed method to count the total number of citations for each use. For sources mentioning the original use reports (UR) for each use and species in the covered territory, we added the UR in the table of results, adding them up. For sources with original verbatim quotations, each quotation referring to the same use was counted as one UR. For sources that did not include the UR for each use (see Table 2 for details), we separately counted each source mentioning this use. Final citations were considered as the sum of the total sources without URs plus the total number of URs from sources with this data. Uses not previously reported also counted as one UR each.

## 5. Conclusions

Information on the wild species traditionally used as food in Andalusia was little known and divulged. The literature review plus a few unpublished data presented here provide data on the edible uses of 336 wild species, representing c. 7% of the total flora of Andalusia. The nutritional potential is known for only a small number of these species. However, the high proportion of species that are also used medicinally by ingesting the same part used as food (24%) is remarkable, and also means that these species can be considered as functional foods for traditional use, which deserves further study as well. Additionally, with few well-known exceptions involving species listed in the conservation laws of the territory or regionally categorised as vulnerable, the rest have no protection nor conservation problems, which supports their traditional and responsible use. Although ethnobotanical studies have already been conducted in several territories of Andalusia, there are still some territorial gaps to be covered by field research. The remarkable cultural and plant diversity of Andalusia, along with its recent history, has led to an intense and diversified exploitation of its wild resources, as presented in this study. The diversity of WEPs is presented as an agroecological solution, either by harvesting species from the wild, or by cultivating them in a low-input cost and drought-resilient type of agriculture.

## Figures and Tables

**Table 1:** WEPs with higher numbers of use reports (UR) and use-mentions in the consulted sources (S).

Scientific Name	S	UR
*Foeniculum vulgare* Mill.	15	90
*Rubus ulmifolius* Schott	13	61
*Scolymus hispanicus* L.	15	58
*Silene vulgaris* (Moench) Garcke	17	57
*Asparagus acutifolius* L.	17	54
*Chamaerops humilis* L.	7	53
*Portulaca oleracea* L.	13	50
*Mentha pulegium* L.	2	49
*Rosmarinus officinalis* L.	13	47
*Silybum marianum* (L.) Gaertner	10	44
*Quercus rotundifolia* Lam.	14	44
*Allium ampeloprasum* L.	12	43
*Thymus mastichina* (L.) L.	11	43
*Arbutus unedo* L.	14	42
*Crataegus monogyna* Jacq.	13	39
*Rorippa nasturtium-aquaticum* (L.) Hayek	14	38
*Anchusa azurea* Mill.	11	35
*Rumex crispus* L.	8	34
*Sonchus oleraceus* L.	13	33
*Opuntia ficus-indica* (L.) Mill.	12	33
*Thymbra capitata* (L.) Cav.	7	28

## Data Availability

All data are available here and in supplementary materials.

## Supplementary Materials

The following supporting information can be downloaded at: https://www.mdpi.com/article/10.3390/plants12061218/s1, Table S1: Potential wild edible plants (PWEPs) of Andalusia.

## Appendix A

WEPs of Andalusia. +: Naturalised plant species. Cat: Category: F: Food; Sn: Snack; Se: Seasoning; D: Drink; Li: Liquor; Cu: Curd. NS: Number of sources citing the plan as WEP in Andalusia. UR: Use reports. Cits: Citations (see Section 4). Sources: see Table 2. ND: New data (previously unpublished). Comments: FCT: Plants with a food composition table (see [7] Cortes Sánchez-Mata and Tardío, 2016); EN: Endangered plants (Decreto 23/2012); VU: Vulnerable; NSA: Need a specific authorization to be gathered in private lands. ND: New data reported here. Refs: 1. Tardío et al., 2006 [35]; 2. Peris et al., 2019 [51]; 3. Rivera et al., 2006b [31]; P: Plants for a future database, [48] PFAF, 2023.

**Family**	**Scientific Name **	**Vernacular Name**	**Medicinal Uses**	**Cat**	**Part Used**	**NS**	**UR**	**Cits**	**Sources**	**Comments**	**Refs. **
Alliaceae	*Allium ampeloprasum* L.	Ajo porro	Digestive disorder; rheuma	F	Bulbs	12	30	42	1, 2, 5, 6, 7, 8, 11, 14, 15, 16, 19, 20	FCT	1; 2; 3; P
				Se	Bulbs	1	0	1	9	Olives dressing	
Alliaceae	*Allium baeticum* Boiss.	Ajo porro, ajo de oso		F	Bulbs	1	0	1	9		
Alliaceae	*Allium neapolitanum* Cirillo	Ajo porro		F	Bulbs	2	0	2	14, 16		1; P
Alliaceae	*Allium roseum* L.	Ajo porro	Digestive disorder	F	Bulbs	3	2	5	1, 12, 13		1; 2; 3; P
Alliaceae	*Allium sphaerocephalon* L.	Ajo porro		F	Bulbs	1	0	1	19		1; P
Alliaceae	*Allium triquetrum* L.	Ajo porro		F	Bulbs	2	0	2	18, 19		2; P
Amaranthaceae	*Amaranthus hybridus* L.	Moco-pavo, bleo, bledo		F	Leaves	1	0	1	20		P
Amaranthaceae	*Amaranthus retroflexus* L.	Moco-pavo, bleo, bledo		F	Leaves	1	1	2	1		P
Anacardiaceae	*Pistacia lentiscus* L.	Lentisco		Se	Leaves	1	0	1	5	NSA; Olives dressing	1; P
Anacardiaceae	*Pistacia terebinthus* L.	Cornicabra		Se	Leaves	1	0	1	16	Olives dressing	P
Aphyllanthaceae	*Aphyllanthes monspeliensis* L.	Junquillo		Sn	Flowers	2	0	2	6, 20		1
Apiaceae	*Apium graveolens* L.	Apio, apio silvestre	Depurative; digestive disorder	F	Leaves	1	0	1	20		1; 2; P
Apiaceae	*Apium nodiflorum* (L.) Lag.	Berra, berro basto		F	Leaves	5	4	9	1, 6, 11, 15, 20	FCT. In certain sources (e.g., 1, 3), toxicity is noted	1; 3
Apiaceae	*Bifora testiculata* (L.) Roth	Culantro		F	Leaves	1	3	4	1		1
				Se	Leaves	3	6	9	1, 8, 9		
Apiaceae	*Bunium macuca* Boiss.	Macuca, amacuca		F	Tubers	5	20	25	1, 3, 6, 14, 20		1; 2
Apiaceae	*Bunium pachypodum* P. W. Ball	Macuca, amacuca		F	Tubers	2	0	2	3, 8		1
Apiaceae	*Bupleurum fruticosum* L.	Crujía, limoncillo		Se	Whole plant	3	0	3	3, 8, 9	Olives dressing: to harder	1
Apiaceae	*Bupleurum gibraltaricum* Lam.	Crujía		Se	Whole plant	4	1	5	1, 3, 8, 9	Olives dressing: to harder	1
Apiaceae	*Conopodium thalictrifolium* (Boiss.) Calest.	Macuca, amacuca		F	Tubers	1	0	1	3		1; 3
Apiaceae	*Crithmum maritimum* L.	Hinojo marino		Se	Leaves	1	0	1	ND	FCT	1; 2; P
				F	Leaves/Stems	1	0	1	ND		
Apiaceae	*Daucus carota* L.	Caíllo, zanahoria silvestre		F	Leaves/Stems	3	1	4	1, 17, 20		1; 2; 3; P
				Sn	Roots	1	1	2	1		
Apiaceae	*Eryngium campestre* L.	Cardo cuco, cardo volador	Circulation problems; digestive disorder; hypertension	F	Roots	1	0	1	1		
				F	Leaves/Stems	3	4	7	11, 13, 14		1; 2; P
				Se	Leaves/Stems	5	0	5	2, 3, 8, 9, 15	Snail dressing in Jaen and Cordoba	
Apiaceae	*Eryngium maritimum* L.	Cardo, quitasueños, hinojo marino		F	Leaves/Stems	1	0	1	19		P
Apiaceae	*Foeniculum vulgare* Mill.	Hinojo	Digestive disorder; gastralgia; flatulence; cold	F	Leaves/Stems	15	39	54	1,3, 4, 5, 6, 7, 8, 10, 11, 13, 14, 16, 17, 20, 21	FCT	1; 2; 3; P
				Se	Leaves	9	17	26	1, 2, 7, 9, 14, 15, 18, 19, 20	NSA; Olives and snail dressing	
				D	Leaves and Fruits	3	2	5	1, 2, 8		
				Sn	Roots/Stems	3	2	5	1, 7, 16		
Apiaceae	*Ridolfia segetum* Moris	Nerdo	Gastralgia; digestive disorder	Se	Leaves	2	2	4	1, 8		
				Cu	Seeds	1	0	1	1		
Apiaceae	*Scandix australis* L.	Quijones, guijones, aguardientera	Gastralgia	F	Whole plant	2	8	10	1, 14		1; 3
				Sn	Aerial parts	3	3	6	1, 3, 14		
				Se	Whole plant	1	0	1	1		
Apiaceae	*Scandix pecten-veneris* L.	Alfilericos, aguardientina, hijones		Sn	Fruits	1	2	3	1		1; 2; 3; P
				F	Leaves	2	1	3	1, 20		
Apiaceae	*Smyrnium olusatrum* L.	Apio caballar, apio silvestre		F	Leaves	3	0	3	5, 7, 17		1; P
Apiaceae	*Thalictrum speciosissimum* L.	Perejil borde		F	Roots	1	0	1	20		
Apiaceae	*Thapsia villosa* L.	Cañaeja	Digestive disorder	F	Roots	1	0	1	1		
Apiaceae	*Torilis nodosa* (L.) Gaertn.	Perejil de campo		Se	Whole plant	1	0	1	15	Traditional dish in Doñana	
Apocynaceae	*Vinca difformis* Pourr.	Campanitas, vinca		Sn	Flowers	1	0	1	3		
Araceae	*Arisarum simorrhinum* Durieu	Candilitos		F	Tubers	1	0	1	19		
Araceae	*Arisarum vulgare* Targ.-Tozz.	Candilicos, zumillo o zomillo		F	Tubers	1	0	1	13		P
Arecaceae	*Chamaerops humilis* L.	Palmito, guaspalma		F	Trunk	7	17	24	1, 11, 12, 13, 15, 19, 21		1; 2; P
				F	Fruits	6	10	16	7, 11, 12, 13, 15, 18	NSA	
				F	Inflorescences	2	11	13	11, 15		
Aristolochiaceae	*Aristolochia baetica* L.	Alcaparrón bravío		F	Tender shoots	1	0	1	15		
Asclepiadaceae	*Periploca angustifolia* Labill.	Cornicabra, cornical, salguilla		Sn	Flowers	1	0	1	13		1
Asparagaceae	*Asparagus acutifolius* L.	Espárrago triguero	Diuretic; kidney malfunction; rheuma; hypercholesterolemia	F	Tender shoots	17	37	54	1, 2, 3, 5, 6, 7, 8, 9, 11, 13, 14, 15, 16, 18, 19, 20, 21	FCT	1; 2; 3; P
Asparagaceae	*Asparagus albus* L.	Espárrago blanco	Diuretic	F	Tender shoots	8	10	18	1, 2, 5, 7, 9, 13, 19, 21		1; 2; P
				Sn	Tender shoots	1	2	3	1		1
Asparagaceae	*Asparagus aphyllus* L.	Espárrago		F	Tender shoots	5	15	20	5, 7, 11, 18, 19		P
Asparagaceae	*Asparagus horridus* L.	Espárrago		F	Tender shoots	4	0	4	3, 5, 13, 14		1; 2
Asparagaceae	*Asparagus officinalis* L. +	Espárrago		F	Tender shoots	1	0	1	ND		1; P
Asteraceae	*Aetheorhiza bulbosa* (L.) Cass.	Castañuela		F	Bulbs	2	2	4	12, 13		1; 2
Asteraceae	*Anacyclus clavatus* (Desf.) Pers.	Manzanilla, mojino blanco, mojigato	Appetizer	F	Leaves/Stems	3	1	4	12, 13, 20		1; 3
				D	Inflorescences	1	0	1	20		
Asteraceae	*Andryala integrifolia* L.	Troncho de vieja, chocho de vieja	Diarrhoea	F	Inflorescences peduncles	2	0	2	9, 19		1
Asteraceae	*Anthemis arvensis* L.	Magarza, manzanilla	Digestive disorder	D	Inflorescences	1	0	1	7		1
Asteraceae	*Arctium minus* (Hill) Bernh.	Bardana, lampazo, arrancamoños, verdelobo		F	Leaves (midrib)	3	1	4	6, 14, 20		1; 2; 3; P
Asteraceae	*Bidens aurea* (Aiton) Sherff +	Té de campo	Digestive disorder	D	Leaves	4	13	17	1, 11, 15, 19		1; P
Asteraceae	*Calendula arvensis* L.	Patagallina, pática de gallo		F	Leaves	1	1	2	13		1; 2; P
Asteraceae	*Calendula officinalis* L. +	Caléndula		F	Leaves/Flowers	1	0	1	ND		2; P
Asteraceae	*Carduncellus caeruleus* (L.) C. Presl	Cepilla		F	Leaves	1	0	1	9		
Asteraceae	*Carthamus tinctorius* L. +	Cártamo, alazor, azafrán falso		Se	Fruits	1	0	1	ND		2; P
Asteraceae	*Centaurea pullata* L.	Escobonera, cepillas		F	Leaves	2	0	2	3, 9		2
Asteraceae	*Cichorium intybus* L.	Achicoria, almirón, camarrojas	Digestive disorder; diuretic; laxative; invigorative	F	Leaves	9	2	11	1, 2, 3, 6, 8, 9, 14, 20, 21	FCT	1; 3; P
				D	Roots	6	2	8	1, 2, 3, 8, 14, 15	Coffee substitute	
				F	Tender shoots	1	6	7	1		
Asteraceae	*Cirsium arvense* (L.) Scop.	Cardo heredero		F	Leaves (midrib)	1	0	1	20		1; P
Asteraceae	*Cirsium rosulatum* Talavera and Valdés	Lechugueta		F	Leaves (midrib)	1	0	1	20		
Asteraceae	*Crepis capillaris* (L.) Wallr.	Almirón		F	Leaves	1	0	1	8		
Asteraceae	*Crepis vesicaria* L. subsp. *taraxacifolia* (Thuill.) Thell.	Almirón		F	Leaves	1	4	5	1		1; 2; P
Asteraceae	*Chamaemelum fuscatum* (Brot.) Vasc.	Manzanilla, clavellina	Digestive disorders	D	Flowered aerial parts	1	0	1	3		1
Asteraceae	*Chondrilla juncea* L.	Chicorias, onjera		F	Leaves	4	4	8	1, 6, 17, 20	FCT	1; 3
Asteraceae	*Chiliadenus glutinosus* (L.) Fourr.	Té de piedra	Digestive disorders	D	Flowered aerial parts	3	4	7	1, 6, 20		1
Asteraceae	*Chrysanthemum coronarium* L.	Crisantemo, mojino amarillo		F	Leaves	1	0	1	13		1; P
Asteraceae	*Cynara cardunculus* L.	Alcacil, alcaucil, alcachofa	Fever	F	Inflorescences/Leaves (midrib)	7	6	13	1, 2, 5, 7, 15, 19, 21		1; 3; P
				Cu	Inflorescences	1	2	3	1		
Asteraceae	*Cynara humilis* L.	Alcacil	Liver conditions	F	Inflorescences	7	0	7	1, 3, 8, 11, 14, 15, 19		1
Asteraceae	*Cynara tournefortii* Boiss. and Reut.	Alcacil, morra		F	Inflorescences	1	0	1	19		
Asteraceae	*Geropogon hybridus* (L.) Sch. Bip.	Teta de vaca		Sn	Roots	1	0	1	3		
Asteraceae	*Glebionis segetum* (L.) Fourr.	Margaritón, crisantemo		F	Leaves	1	0	1	ND		
Asteraceae	*Hedypnois rhagadioloides* (L.) F.W. Schmidt	Mata dulce		F	Leaves	1	0	1	13		1; 2
Asteraceae	*Helianthus tuberosus* L. +	Papa de caña, papa de sierra		F	Tubers	2	5	7	1, 2		1; 2; P
Asteraceae	*Helichrysum italicum* (Roth.) G. Don fil.	Manzanilla amarga, monte chivero	Digestive disorders	D	Flowered aerial parts	1	0	1	11	NSA	1; P
Asteraceae	*Helichrysum stoechas* (L.) Moench	Manzanilla amarga	Digestive disorders	D	Flowered aerial parts	1	4	5	11	NSA	1
Asteraceae	*Hypochaeris radicata* L.	Almirón, tetas de vaca		F	Stems	1	0	1	3		1; 2; 3; P
Asteraceae	*Lactuca saligna* L.	Chicoria, moquillo, forraja, lechugueta		F	Leaves/Stems	3	0	3	3, 6, 20		1
Asteraceae	*Lactuca serriola* L.	Lechugueta		F	Leaves/Stems	4	3	7	1, 8, 14, 20		1; P
Asteraceae	*Lactuca singularis* Wilmott	Moquillo		F	Leaves	1	0	1	17		
Asteraceae	*Lactuca tenerrima* Pourr.	Pan de pobre, lechugón, lechugueta		F	Leaves/Stems	4	2	6	1, 6, 10, 20		1; 3
Asteraceae	*Lactuca viminea* (L.) F.W. Schmidt	Achicoria		F	Leaves/Stems	1	0	1	3		1
Asteraceae	*Leontodon longirrostris* (Finch and P. D. Sell) Talavera	Almirones		F	Leaves	1	1	2	1		1; 2
Asteraceae	*Leontodon tuberosus* L.	Almirones		F	Leaves/Roots	1	0	1	ND		1; 2
Asteraceae	*Leuzea conifera* (L.) DC.	Alcachofilla, alcancilillo, cepilla		F	Inflorescences	4	1	5	2, 6, 9, 20		1
Asteraceae	*Mantisalca salmantica* (L.) Briq. and Cavillier	Rama, escobicas, escobón, pan de pastor	Hyperglucemia	F	Leaves	6	5	11	1, 2, 6, 8, 17, 14		1; 2; 3
Asteraceae	*Matricaria chamomilla* L.	Manzanilla	Gastralgia; digestive disorder; dysmenorrhoea; cold; cough; flatulence; female genital infection	D	Flowered aerial parts	4	1	5	1, 6, 15, 20		1; P
				Li	Flowered aerial parts	1	1	2	1		
Asteraceae	*Onopordum acanthium* L.	Cardo borriquero, toba		F	Leaves midrib/Stems	1	0	1	ND		1; 3; P
Asteraceae	*Onopordum macracanthum* Schousb.	Cardo, cardoncha, pincho burrero		F	Tender shoots/Stems	2	3	5	12, 13		1
Asteraceae	*Onopordum nervosum* Boiss.	Toba		F	Leaves	2	2	4	1, 14		1; 3
Asteraceae	*Picris echioides* L.	Chicoria, lengua de vaca		F	Tender shoots	1	0	1	8		1; 2; P
Asteraceae	*Picnomon acarna* (L.) Cass.	Cardo blanco		F	Tender shoots	2	0	2	6, 20		
Asteraceae	*Rhagadiolus edulis* Gaertn.	Clica		F	Leaves	1	4	5	ND	Granada	2; 3
Asteraceae	*Santolina rosmarinifolia* L. subsp. *canescens* (Lag.) Nyman	Meaperros		Se	Flowered aerial parts	1	0	1	1	NSA; Olives dressing	1; P
Asteraceae	*Scolymus hispanicus* L.	Cardillo, tagarninas, cardo santo, cardocristo		F	Leaves/Stems (midrib)	15	43	58	1, 2, 3, 4, 5, 7, 11, 12, 14, 15, 16, 17, 18, 19, 21	FCT	1; 3; P
Asteraceae	*Scolymus maculatus* L.	Cardillo, tagarninas		F	Leaves/Stems (midrib)	5	4	9	1, 6, 9, 15, 19		1; 2; P
Asteraceae	*Scorzonera angustifolia* L.	Tetilla, tetilla de vaca		Sn	Roots	2	4	6	1, 3		1; 3
				F	Leaves/Stems	1	0	1	8		
Asteraceae	*Scorzonera hispanica* L.	Escarcionera, tetilla de vaca		Sn	Roots	2	6	8	1, 3		1; 2; P
Asteraceae	*Scorzonera laciniata* L.	Tetilla de vaca		Sn	Roots	2	1	3	1, 3		1; 3
				Sn	Inflorescences peduncles	2	0	2	14, 15		
Asteraceae	*Silybum marianum* (L.) Gaertner	Cardo borriquero	Liver disease; Malta fever	F	Inflorescences/Leaves (midrib)	10	27	37	1, 2, 3, 5, 6, 7, 9, 11, 14, 15	FCT	1; 2; 3; P
				Cu	Inflorescences	3	1	4	1, 3, 6		
				Sn	Stems	2	1	3	1, 11		
Asteraceae	*Sonchus asper* (L.) Hill.	Cerraja, diente de león		F	Leaves	3	0	3	2, 3, 16	FCT	1; 3; P
Asteraceae	*Sonchus oleraceus* L.	Cerraja, cerrajón		F	Leaves	13	20	33	1, 3, 5, 6, 7, 8, 11, 12, 13, 14, 15, 16, 20	FCT	1; 3; P
Asteraceae	*Sonchus tenerrimus* L.	Cerraja		F	Leaves	1	3	4	12		1; 3; P
Asteraceae	*Taraxacum erythrospermum* Andrz. ex Besser	Almirón, tetas de vaca		F	Leaves	2	0	2	1, 6		1;
				D	Leaves	1	0	1	2	Coffee substitute	
Asteraceae	*Taraxacum obovatum* (Willd.) DC.	Diente de león, flor de pitón, pitones		F	Leaves	1	0	1	6	FCT	1; 2; 3; P
Asteraceae	*Taraxacum vulgare* (Lam.) Schrank	Almiron, diente de león	Asthenia; kidney and liver disease; depurative; laxative	F	Aerial parts	1	3	4	1	FCT	1; 3
				D	Inflorescences	1	0	1	2		
Asteraceae	*Tragopogon crocifolius* L.	Teticas de vaca		Sn	Roots/Stems	1	9	10	1		3; P
Asteraceae	*Tragopogon porrifolius* L.	Tetillón, teticas de vaca, toba		Sn	Roots/Stems	4	0	4	1, 3, 6, 14		1; 3; P
Asteraceae	*Urospermum picroides* (L.) Scop	Cerraja, clavellina.		F	Leaves	1	0	1	3		1; 2
Betulaceae	*Corylus avellana* L.	Avellano		F	Fruits	4	1	5	6, 8, 14, 20		1; P
Berberidaceae	*Berberis hispanica* Boiss. and Reut.	Agracejo	Kidney malfunction	F	Fruits	3	6	9	1, 6, 20		1; 3
Boraginaceae	*Alkanna tinctoria* (L.) Tausch	Raíz de palomino		Se	Roots	1	0	1	17	For food colouring	P
Boraginaceae	*Anchusa azurea* Mill.	Lenguaza	Kidney stones	F	Leaves/Stems	11	19	30	1, 2, 3, 5, 6, 8, 9, 16, 17, 20, 21	FCT	1; 3; P
				F	Roots	1	0	1	14		
				Sn	Flowers	3	1	4	1, 3, 11		
Boraginaceae	*Anchusa undulata* L. subsp. *granatensis* (Boiss.) Valdés	Lenguaza		F	Leaves/Stems	2	0	2	1, 20		1
Boraginaceae	*Borago officinalis* L. +	Borraja, almorraza	Digestive disorder	F	Leaves/Stems	10	5	15	1, 3, 5, 7, 9, 11, 14, 15, 16, 21	FCT	1; 2; P
				Sn	Flowers	2	1	3	1, 11		
Boraginaceae	*Echium creticum* L. subsp. *granatense* (Coincy) Valdés	Falsa lenguaza		F	Stems	2	0	2	1, 9		1
Brassicaceae	*Alliaria petiolata* (M. Bieb.) Cavara and Grande	Ajera, hierba del ajo		F	Leaves	1	0	1	ND		P
Brassicaceae	*Brassica nigra* (L.) Koch	Mostaza		F	Leaves	1	0	1	ND		P
				Se	Seeds	1	0	1	ND		
Brassicaceae	*Capsella bursa-pastoris* L.	Bolsa de pastor, pan y quesillo, raserica		F	Leaves	2	0	2	6, 20	FCT	1; 2; 3; P
Brassicaceae	*Cardaria draba* (L.) Desv.	Flor de muerto, achicoria		F	Leaves	1	0	1	ND		2; P
				D	Whole plant	1	0	1	4	Coffee substitute	
Brassicaceae	*Diplotaxis erucoides* (L.) DC.	Rabaniza, oruga		F	Leaves	1	0	1	ND		1; 2; P
Brassicaceae	*Eruca vesicaria* (L.) Cav.	Jaramago, jaramago blanco		F	Leaves	4	2	6	3, 13, 14, 16	Including *E. sativa* Mill. FCT	1; 2; 3; P
Brassicaceae	*Hirschfeldia incana* (L.) Lagr.–Foss.	Jaramargo		F	Stems	1	1	2	13		1; P
Brassicaceae	*Moricandia arvensis* (L.) DC.	Collejón, collejón basto		F	Leaves	1	0	1	14		1
Brassicaceae	*Raphanus sativus* L. +	Rabaneta, rábano silvestre		F	Leaves	5	0	5	5, 7, 8, 14, 20		P
Brassicaceae	*Rapistrum rugosum* (L.) All.	Jaramago, amargo, rábano		F	Leaves	2	0	2	8, 20		1
Brassicaceae	*Rorippa nasturtium-aquaticum* (L.) Hayek	Berro, mastuerzo		F	Leaves/Stems	14	24	38	1, 2, 3, 5, 6, 7, 8, 11, 14, 15, 16, 19, 20, 21	FCT	1; 2; 3; P
Brassicaceae	*Sinapis alba* subsp. *mairei* (H. Lindb.) Maire	Jaramago	Liver disease; menorrhagia	F	Leaves	3	2	5	1, 3, 8		1; P
				Se	Seeds	1	0	1	ND		
Cactaceae	*Opuntia ficus-indica* (L.) Mill. +	Chumbera, penca	Digestive disorder; diarrhoea; cold; cough	F	Fruits	12	20	32	1, 2, 3, 5, 7, 8, 11, 14, 15, 19, 20, 21		1; 2; P
				D	Leaves	1	0	1	15	Direct consumption of inner leaf water	
Cactaceae	*Opuntia megacantha* Salm-Dyck +	Chumbera		F	Fruits	1	0	1	19		
Cactaceae	*Opuntia tuna* (L.) Mill. +	Chumbera		F	Fruits	1	0	1	19		
Campanulaceae	*Campanula rapunculus* L.	Raponchigo		F	Roots	1	0	1	16		1; 2; P
Capparidaceae	*Capparis spinosa* L.	Alcaparra	Appetizer	F	Flowers/Fruits	9	11	20	1, 2, 3, 5, 8, 9, 13, 14, 20		1; 2; P
Caprifoliaceae	*Lonicera etrusca* G. Santi	Mariselva, madreselva		Sn	Flowers	1	1	2	1		
Caprifoliaceae	*Lonicera implexa* Aiton	Mariselva, madreselva		Sn	Flowers	1	1	2	1		1
Caprifoliaceae	*Lonicera japonica* L. +	Madreselva		Sn	Flowers	1	0	1	14		P
Caprifoliaceae	*Lonicera periclymenum* L.	Mariselva, madreselva		Sn	Flowers	1	0	1	ND		1
Caprifoliaceae	*Lonicera splendida* Boiss.	Mariselva, madreselva		Sn	Flowers	1	0	1	ND		
Caprifoliaceae	*Sambucus nigra* L.	Saúco, sabuco		F	Fruits	1	0	1	ND		1; P
Caprifoliaceae	*Viburnum tinus* L.	Durillo		Se	Whole plant	1	0	1	5	Olives dressing	
Caryophyllaceae	*Silene vulgaris* (Moench) Garcke	Collejas		F	Leaves/Stems	17	40	57	1, 2, 3, 4, 5, 6, 7, 8, 9, 10, 11, 14, 15, 16, 17, 19, 21	FCT	1; 2; 3; P
Caryophyllaceae	*Stellaria media* (L.) Vill.	Pamplina		F	Whole plant	1	0	1	3		1
Caryophyllaceae	*Stellaria pallida* (Dumort.) Piré	Pamplina		F	Whole plant	2	0	2	3, 20		
Caryophyllaceae	*Vaccaria hispanica* (Miller) Rauschert	Hiel de la tierra		F	Leaves	1	1	2	1		1; P
Chenopodiaceae	*Arthrocnemum macrostachyum* (Moric.) Moris	Almajo salado, sosa jabonera		F	Leaves	1	0	1	21		
Chenopodiaceae	*Atriplex prostrata* DC.	Espinaca		F	Leaves	1	0	1	3		1
Chenopodiaceae	*Beta vulgaris* L. +	Remolacha		F	Leaves	1	0	1	ND		2
Chenopodiaceae	*Beta maritima* L.	Acelga silvestre, acelguilla, penca	Digestive problems	F	Leaves	6	17	23	6, 11, 12, 14, 15, 20	FCT	1; 2; 3; P
Chenopodiaceae	*Chenopodium album* L.	Cenizo		F	Leaves/Seeds	2	0	2	16, 19	FCT	1; 3; P
Chenopodiaceae	*Chenopodium murale* L.	Corralera		F	Leaves	1	0	1	19		1; P
Chenopodiaceae	*Chenopodium opulifolium* Koch and Ziz	Cenizo		F	Leaves	1	0	1	19		P
Chenopodiaceae	*Salsola vermiculata* L.	Barrilla, sosa, patagusano		Se	Stems	1	0	1	13	To make chickpeas softer	
Chenopodiaceae	*Sarcocornia fruticosa* (L.) A. J. Scott	Sapina, almajo salao		F	Stems	1	0	1	21		
Chenopodiaceae	*Suaeda vera* J. F. Gmelin	Sosa, almajo dulce		F	Stems	1	0	1	21		
Colchicaceae	*Colchicum lusitanicum* Brot.	Azafrán silvestre		Se	Stigma	1	0	1	3	Saffron substitute	
Convolvulaceae	*Convolvulus althaeoides* L.	Corregüela basta		Sn	Flowers	1	0	1	14		
Convolvulaceae	*Convolvulus arvensis* L.	Corregüela, correvuela, campanillas		F	Leaves	1	0	1	20		1; P
Crassulaceae	*Pistorinia hispanica* (L.) DC.	Vinagreta		F	Leaves	1	0	1	20		
Crassulaceae	*Umbilicus gaditanus* Boiss.	Ombligo de Venus, ombliguito, sombrerillo		Sn	Leaves	2	0	2	18, 19		
Crassulaceae	*Umbilicus rupestris* (Salisb.) Dandy	Ombligo de Venus, ombliguito, sombrerillo		Sn	Leaves	2	0	2	18, 19		P
Crassulaceae	*Sedum sediforme* (Jacq) Pau.	Uña de gato		Sn	Leaves	1	2	3	1		2; P
Cupressaceae	*Juniperus communis* L.	Enebro		Li	Fruits	1	0	1	20		1; 3; P
Cupressaceae	*Juniperus oxycedrus* L.	Enebro		Se	Aerial parts/Fruits	1	0	1	5	Cheese flavouring	1; 3
				Li	Fruits	2	0	2	15, 20	To make local gin	
Cupressaceae	*Juniperus sabina* L.	Enebro		Li	Fruits	1	0	1	20		
Cyperaceae	*Bolboschoenus maritimus* (L.) Palla	Juncia		F	Stems/Rhizomes	2	1	3	11, 15		
Cyperaceae	*Cyperus longus* L.	Juncia		F	Rhizomes	1	0	1	20		P
Cyperaceae	*Cyperus rotundus* L.	Castañuela		F	Stems/Rhizomes	2	6	8	11, 15		P
Cyperaceae	*Schoenoplectus lacustris* (L.) Palla	Bayuncos		F	Stems/Rhizomes	1	0	1	15		
Cyperaceae	*Scirpoides holoschoenus* (L.) Sojak	Junco	Cold; cough; appetizer	Sn	Leaves	8	7	15	1, 3, 5, 6, 8, 9, 14, 20		1; 2
Diocoreaceae	*Tamus communis* L.	Espárragos de bicha, espárragos de culebra, esparraguina		F	Tender shoots	6	0	6	3, 5, 7, 14, 15, 21	FCT	1; 2; P
Empetraceae	*Corema album* (L.) D. Don	Camarina		F	Fruits	2	16	18	11, 15		P
Ericaceae	*Arbutus unedo* L.	Madroño		F	Fruits	14	22	36	1, 2, 3, 5, 6, 7, 8, 11, 14, 15, 16, 19, 20, 21	NSA; FCT	1; 3; P
				Li	Fruits	3	1	4	1,2, 7		
				Se	Aerial parts	2	0	2	7, 14	Olives dressing	
Euphorbiaceae	*Euphorbia characias* L.	Lecheinterna, lechetrezna		Cu	Latex	1	0	1	14		1
Euphorbiaceae	*Euphorbia nicaeensis* All.	Lecheterna, lecheinterna, lechetrezna, reicheruela		Cu	Latex	1	0	1	14		1
Euphorbiaceae	*Euphorbia serrata* L.	Lecheterna, lecheinterna, lechetrezna, reicheruela		Cu	Whole plant/Latex	3	0	3	6, 13, 20		1; 3
Fabaceae	*Ceratonia siliqua* L.	Algarrobo	Diarrhoea; haemorrhoids	F	Fruits	9	6	15	1, 3, 5, 7, 8, 9, 13, 14, 19		1; P
				F	Seeds	1	0	1	7	Sugar substitute	
				Sn	Fruits	1	0	1	14		
Fabaceae	*Cytisus scoparius* (L.) Link	Hiniesta, retama negra		F	Flowers	1	0	1	20	For dying Eastern eggs	P
				F	Fruits	1	0	1	20		
Fabaceae	*Gleditsia triacanthos* L. +	Algarrobo bravío		Sn	Fruits	2	1	3	1, 5		P
Fabaceae	*Glycyrrhiza glabra* L.	Regaliz	Cold	Sn	Roots	4	21	25	1, 2, 11, 14		1; 3; P
Fabaceae	*Lathyrus cicera* L.	Almorta silvestre		Sn	Seeds	1	0	1	ND		1; 3; P
Fabaceae	*Lathyrus sativus* L.	Guijas, almorta		F	Seeds	1	0	1	14		1; 3; P
Fabaceae	*Lathyrus clymenum* L.	Guajas, présule zorrero		F	Fruits	1	1	2	13		1
Fabaceae	*Lupinus albus* L. +	Altramuz		Sn	Seeds	1	0	1	ND		P
Fabaceae	*Medicago sativa* L.+	Alfalfa, mielga	Hypercholesterolemia; hyperglucemia; kidney malfunction; rheuma	F	Aerial parts	2	3	5	1, 2		1; 2; P
Fabaceae	*Robinia pseudoacacia* L. +	Acacia		Sn	Flowers	6	3	9	1, 2, 3, 5, 18, 19		1; 3; P
Fabaceae	*Scorpiurus muricatus* L.	Orejas de liebre		F	Leaves	5	1	6	6, 8, 12, 13, 20		1; 2; P
Fabaceae	*Vicia cordata* Hoppe	Arvejana, albejana, vesa		F	Seeds	1	0	1	8		
Fabaceae	*Vicia tenuifolia* Roth	Arvejana		F	Seeds	1	0	1	14		P
Fabaceae	*Vicia peregrina* L.	Grisoles		F	Seeds	2	0	2	1, 20		1
Fabaceae	*Vicia sativa* L. +	Veza		F	Fruits	4	1	5	1, 13, 14, 19		1; P
Fagaceae	*Castanea sativa* Mill. +	Castaño		F	Fruits	1	0	1	ND		1; P
Fagaceae	*Quercus coccifera* L.	Coscoja		F	Fruits	2	1	3	1, 20	Soaked in water to remove bitterness	1; P
Fagaceae	*Quercus rotundifolia* Lam.	Encina	Diarrhoea; diarrhoea with tenesmus; gastralgia	F	Fruits	14	28	42	1, 2, 5, 6, 7, 8, 9, 11, 14, 15, 16, 17, 19, 20		1; 3; A
				Sn	Leaves	1	1	2	1		
Geraniaceae	*Erodium ciconium* (L.) L’Hér.	Alfilerillos, relojes		Sn	Fruits	1	0	1	3		1
Geraniaceae	*Erodium cicutarium* (L.) LHér.	Alfilericos		F	Leaves	1	4	5	1		1; P
				Sn	Fruits	2	0	2	3, 14		
Geraniaceae	*Erodium malacoides* (L.) L’Hér.	Alfilerillos, relojes		Sn	Fruits	3	0	3	3, 8, 14		1; P
Geraniaceae	*Erodium moschatum* (L.) L’Hér.	Alfilerillos, relojes		Sn	Fruits	2	0	2	3, 14		P
Geraniaceae	*Erodium primulaceum* Welw. ex Lange	Alfilerillos, relojes		Sn	Fruits	1	0	1	3		
Grossulariaceae	*Ribes alpinum* L.	Grosellero		F	Fruits	1	0	1	2	VU	1; P
Grossulariaceae	*Ribes uva-crispa* L.	Grosellero		F	Fruits	1	0	1	2	VU	1; 3; P
Hyacinthaceae	*Muscari neglectum* Guss.	Nazareno, moreta		F	Bulbs	2	1	3	6, 20		1; P
Hypolepidaceae	*Pteridium aquilinum* (L.) Kuhn	Helecho		F	Tender shoots	2	0	2	18, 19		P
Iridaceae	*Crocus serotinus* Salisb. *subsp. salzmannii* (J. Gay) Mathew	Azafrán silvestre		Se	Stigma	1	0	1	3	Saffron substitute	1; P
Iridaceae	*Crocus nevadensis* Amo	Azafrán silvestre		Se	Stigma	1	0	1	ND	Saffron substitute	1; 3
Iridaceae	*Gladiolus communis* L.	Española, gladiolo, palmito, pintauñas		F	Bulbs	2	0	2	3, 8		
Iridaceae	*Gladiolus italicus* Mill.	Española, gladiolo, palmito, pintauñas		F	Bulbs	1	0	1	3		
Iridaceae	*Gynandriris sisyrinchium* (L.) Parl.	Patita de burro		F	Bulbs	2	1	3	11, 15		P
Iridaceae	*Romulea ramiflora* Ten.	Cutufa, castañuela		F	Bulbs	1	0	1	19		
Juglandaceae	*Juglans regia* L. +	Nogal		F	Seeds	12	3	15	1, 2, 4, 5, 6, 7, 8, 9, 13, 14, 15, 20		1; 3; P
Lamiaceae	*Acinos alpinus* (L.) Moench	Té de la sierra, té	Digestive; genito-urinary analgesic; appetizer	D	Flowered aerial parts	2	2	4	1, 20	NSA	1; P
Lamiaceae	*Calamintha nepeta* (L.) Savi	Neota, hierba pastora		Se	Flowered aerial parts	1	0	1	2		1; P
				D	Flowered aerial parts	1	0	1	19		
Lamiaceae	*Lavandula dentata* L.	Cantueso		Se	Leaves	1	2	3	ND	NSA; Olives dressing	1
Lamiaceae	*Lavandula lanata* Boiss.	Alhucemón		Se	Flowered aerial parts	1	0	1	ND	NSA	
Lamiaceae	*Lavandula latifolia* L.	Alhucema	Circulatory disorders; digestive; cold; cough	Se	Leaves	3	0	3	6, 14, 20	NSA; Olives dressing	1; P
Lamiaceae	*Lavandula stoechas* L.	Cantueso	Diabetes; digestive disorder; cold	Se	Leaves	1	2	3	1	NSA; Olives dressing	1
Lamiaceae	*Melissa officinalis* L. +	Melisa, torojil	Circulatory problems; nervousness; diarrhoea	D	Flowered aerial parts	1	1	2	1		1; P
				Li	Leaves	1	0	1	2		
				Se	Aerial parts	1	0	1	13		
Lamiaceae	*Mentha aquatica* L.	Menta		Se	Leaves	1	0	1	ND		1; P
Lamiaceae	*Mentha longifolia* (L.) Huds.	Menta caballar, mastranzo		Se	Leaves	1	0	1	ND		1; P
				D	Flowered aerial parts	1	0	1	20		
Lamiaceae	*Mentha pulegium* L.	Poleo	Digestive disorder; gastralgia; dysmenorrhoea; circulatory problems; cough; kidney stones; postpartum infections	Li	Flowered aerial parts	2	4	6	1, 2		1; P
				Se	Flowered aerial parts	6	17	23	2, 11, 15, 16, 19, 21	NSA	
				D	Flowered aerial parts	3	17	20	3, 11, 15		
Lamiaceae	*Mentha spicata* L. +	Hierbabuena	Digestive disorder; gastralgia; cough	Se	Aerial parts	3	0	3	2, 14, 15		1; 2; 3; P
Lamiaceae	*Mentha suaveolens* Ehrh.	Mastranzo, mestranzo	Stomach or intestinal problems; intestinal parasites	Se	Leaves/Stems	6	3	9	6, 9, 13, 14, 16, 20		1; P
Lamiaceae	*Mentha piperita* L. +	Menta piperita, hierbabuena	Intestinal parasites; appetizer	Li	Leaves	2	0	2	2, 4		P
				Se	Leaves	6	4	10	4, 8, 9, 13, 19, 20		
				D	Flowered aerial parts	1	2	3	20		
Lamiaceae	*Origanum compactum* Benth.	Orégano		Se	Flowered aerial parts	1	0	1	19	Olives dressing	P
Lamiaceae	*Origanum virens* Hoffmanns and Link	Orégano	Cold; cough; toothache; digestive disorder	Se	Flowered aerial parts	11	7	18	1, 2, 3, 5, 6, 8, 9, 14, 18, 19, 21	NSA; Olives dressing	1; 2; 3; P
Lamiaceae	*Phlomis lychnitis* L.	Candilera, matagallo real	Gallbladder and kidney stones; stomach problems; diuretic; hypercholesterolemia	Sn	Flowers	1	0	1	3		1; P
Lamiaceae	*Phlomis purpurea* L.	Matagallos		Sn	Flowers	1	0	1	3		1
Lamiaceae	*Rosmarinus officinalis* L.	Romero	Cold; cough; mouth infections; circulatory problems; gastritis; bronchitis; appetizer	Se	Leaves	13	31	44	1, 2, 4, 5, 8, 11, 13, 14, 15, 16, 19, 20, 21	NSA; Olives dressing	1; 2; 3; P
				D	Flowered aerial parts	3	0	3	2, 11, 20		
Lamiaceae	*Salvia lavandulifolia* Vahl	Salvia	Digestive disorder; circulatory problems; cold	Se	Leaves	1	1	2	1	NSA; Olives dressing	1; 3; P
				D	Flowered aerial parts	1	2	3	1		
Lamiaceae	*Salvia microphylla* Kunth +	Flor de mixto		Sn	Flowers	2	6	8	1, 3		P
				D	Flowered aerial parts	1	0	1	1		
Lamiaceae	*Salvia verbenaca* L.	Crestagallo		F	Leaves	1	0	1	20		P
Lamiaceae	*Satureja intricata* Lange	Tomillo		Se	Leaves	1	0	1	2	NSA; Olives dressing	1; 3
Lamiaceae	*Satureja obovata* Lag.	Tomillo		Se	Leaves	5	18	23	1, 2, 3, 8, 13	NSA; Olives dressing	1; 3
Lamiaceae	*Teucrium aureum* Schreb.	Zamarrilla blanca o fina	Appetizer	D	Flowered aerial parts	1	0	1	20		
Lamiaceae	*Thymbra capitata* (L.) Cav.	Tomillo blanco	Cold; inflammation	Se	Flowered aerial parts	7	20	27	1, 3, 8, 9, 11, 15, 18	NSA; Olives dressing	2; P
				D	Flowered aerial parts	1	0	1	11		
Lamiaceae	*Thymus baeticus* Lacaita	Tomillo limonero, fino		Se	Flowered aerial parts	1	0	1	ND	NSA; Olives dressing	1
Lamiaceae	*Thymus hyemalis* Lange	Tomillo, tomillo negro, salsero, o colorao		Se	Flowered aerial parts	1	10	11	13	NSA; Olives dressing	1
Lamiaceae	*Thymus longiflorus* Boiss.	Tomillo real		Se	Flowered aerial parts	1	2	3	1		
Lamiaceae	*Thymus mastichina* (L.) L.	Almoradúx, marabú, mejorana, tomillo borriquero	Cold; digestive disorder; gastralgia; bronchitis; urinary infection	Se	Flowered aerial parts	11	16	27	1, 2, 3, 4, 5, 9, 11, 14, 15, 20, 21	NSA; Olives dressing	1; P
				D	Flowered aerial parts	4	9	13	2, 11, 15, 20		
				Li	Flowered aerial parts	2	1	3	1, 2		
Lamiaceae	*Thymus orospedanus* Huguet del Villar	Tomillo basto	Appetizer	Se	Flowered aerial parts	4	5	9	1, 2, 6, 20	Olives dressing	1
Lamiaceae	*Thymus serpylloides* Bory subsp. *serpylloides*	Tomillo de la sierra		Se	Flowered aerial parts	1	0	1	ND		1
Lamiaceae	*Thymus serpylloides* Bory subsp. *gadorensis* (Pau) Jalas	Tomillo de la sierra		Se	Flowered aerial parts	1	0	1	ND		1
Lamiaceae	*Thymus vulgaris* L.	Tomillo	Same as for *T. zygis*	Se	Flowered aerial parts	1	0	1	14	NSA; Olives dressing	1; 2; 3; P
Lamiaceae	*Thymus zygis* Loefl. ex L. subsp. *gracilis* (Boiss.) R. Morales	Tomillo aceitunero	Cold; urinary infection; digestive disorder; infection; cough; hypercholesterolemia; depurative	Se	Flowered aerial parts	6	16	22	1, 2, 3, 4, 5, 14	NSA; Olives dressing	1; 2; 3; P
Lamiaceae	*Ziziphora hispanica* L.	Poleillo		D	Flowered aerial parts	1	0	1	ND		1
Lauraceae	*Laurus nobilis* L. +	Laurel	Flatulence	Se	Leaves	5	11	16	1, 8, 9, 14, 18	Olives dressing	1; P
Malvaceae	*Lavatera cretica* L.	Malva, panecillos		Sn	Flowers/Fruits	6	14	20	1, 2, 3, 6, 11, 20		
				F	Leaves	1	1	2	3		1; 2
Malvaceae	*Malva aegyptia* L.	Malva		Sn	Fruits	2	0	2	6, 20		
Malvaceae	*Malva neglecta* Wallr.	Malva		Sn	Fruits	2	0	2	6, 20		1; P
Malvaceae	*Malva nicaeensis* All.	Malva		Sn	Fruits	4	6	10	1, 3, 6, 20		1; P
				F	Leaves	1	1	2	3		
Malvaceae	*Malva parviflora* L.	Malva	Haemorrhoids	Sn	Fruits	4	0	4	2, 6, 14, 20		1; P
				F	Leaves	1	1	2	3		
Malvaceae	*Malva sylvestris* L.	Malva		Sn	Flowers/Fruits	8	6	14	1, 3, 5, 6, 7, 8, 14, 19	FCT	1; 2; 3; P
				F	Leaves	7	1	8	3, 5, 7, 8, 13, 14, 16		
Moraceae	*Ficus carica* L.	Higuera	Cold; cough; constipation; liver disease	F	Fruits	9	11	20	1,2, 5, 7, 8, 13, 14, 19, 20		P
				Cu	Leaves/Latex	3	1	4	1, 8, 14		
				Se	Fruits	1	0	1	14	To make vinegar	
Moraceae	*Morus alba* L.	Moral		F	Fruits	4	0	4	3, 5, 7, 14		P
Moraceae	*Morus nigra* L.	Morera		F	Fruits	4	0	4	3, 6, 14, 20		P
				Li	Fruits	1	0	1	3		
Myrtaceae	*Eucalyptus camaldulensis* Dehnh.	Eucalipto	Cold; cough; bronchitis; hyperglucemia	Se	Leaves	1	0	1	20		P
Myrtaceae	*Myrtus communis* L.	Arrayán, mirto		F	Fruits	3	9	12	11, 15, 18	FCT	1; P
				Se	Leaves	4	0	4	3, 5, 8, 15	NSA; Olives dressing	
				Li	Fruits	1	0	1	16		
Oleaceae	*Olea europaea* L. var. *sylvestris* Brot.	Olivo		F	Fruits	6	8	14	1, 2, 7, 11, 15,20	Oil is made and fruits are seasoned	1; 2;
				Se	Leaves	1	0	1	5	Olives dressing	
Oxalidaceae	*Oxalis debilis* Kunth	Trébol		Sn	Leaves	1	0	1	14		
Oxalidaceae	*Oxalis corniculata* L.	Trébol, vinagreta		Sn	Leaves	1	0	1	ND		P
Oxalidaceae	*Oxalis latifolia* Kunth	Trébol		Sn	Leaves	1	0	1	14		1
Oxalidaceae	*Oxalis pes-caprae* L.	Vinagrera, vinagretas, vinagritos		Sn	Flowers	2	2	4	11, 13		1; 2; P
				F	Rhizomes	2	1	3	12, 13		
				F	Leaves	3	0	3	16, 18, 19		
Papaveraceae	*Fumaria parviflora* Lam.	Zapaticos		Sn	Flowers	1	1	2	1		
Papaveraceae	*Papaver rhoeas* L.	Amapola		F	Leaves	6	2	8	1, 5, 7, 13, 14, 16	FCT	1; 2; 3; P
				F	Seeds	1	0	1	7		
Papaveraceae	*Papaver somniferum* L.	Amapola real, adormidera		F	Seeds	1	0	1	ND	Bakery	P
Pinaceae	*Pinus halepensis* Mill.	Pino		Sn	Seeds	2	0	2	14, 20		1; P
Pinaceae	*Pinus pinea* L. +	Pino piñonero		F	Seeds	9	18	27	1, 5, 6, 7, 11, 14, 15, 19, 20		1; 2; 3; P
Plantaginaceae	*Plantago albicans* L.	Pan de pastor, hierba yesquera	Diarrhoea	F	Leaves	2	1	3	9, 13		1
Plantaginaceae	*Plantago lanceolata* L.	Llantén	Digestive disorders	F	Leaves	1	0	1	ND	FCT	1; 2; P
Plantaginaceae	*Plantago major* L.	Llantén mayor	Digestive disorders	F	Leaves	1	0	1	16	FCT; NSA	1; 2; P
Plantaginaceae	*Plantago media* L.	Llantén	Digestive disorders	F	Leaves	1	0	1	ND		P
Plumbaginaceae	*Limonium sinuatum* (L.) Mill.	Capitana, siempreviva		F	Leaves/Stems	2	4	6	12, 13	NSA	1
Poaceae	*Aegilops geniculata* Roth	Rompesacos, rompisacos		F	Seeds	1	1	2	9		1; 3
				Sn	Seeds	1	0	1	14		
Poaceae	*Aegilops triuncialis* L.	Rompisacos		Sn	Seeds	1	0	1	14		1; P
Poaceae	*Arundo donax* L. +	Caña		F	Rhizomes	1	2	3	1		1; P
Poaceae	*Avena sterilis* L.	Avena borde		F	Seeds	1	0	1	20		P
Poaceae	*Cynodon dactylon* (L.) Pers.	Grama	Diuretic; appetizer	F	Rhizomes	4	2	6	5, 7, 13, 20		1; 3; P
Poaceae	*Dactylis glomerata* L.	Triguera		Sn	Inflorescences peduncles	1	0	1	20		
Poaceae	*Hordeum murinum* L. subsp. *leporinum* (Link) Arcang.	Ballico, cebada		Sn	Inflorescences peduncles	1	0	1	ND		P
				F	Seeds	1	0	1	20		
Poaceae	*Hordeum murinum* L. subsp. *murinum*	Cebailla		Sn	Inflorescences peduncles	1	0	1	14		P
Poaceae	*Macrochloa tenacissima* (L.) Kunth	Esparto		Sn	Inflorescences peduncles	2	0	2	1, 19	NSA	
Poaceae	*Neoschischkinia nebulosa* (Boiss. and Reut.) Tzvelev	Barresantos		Sn	Inflorescences peduncles	1	0	1	20		
Poaceae	*Polypogon monspeliensis* (L.) Desf.	Té	Appetizer	D	Aerial parts	1	0	1	20		P
Poaceae	*Sorghum halepense* (L.) Pers.	Sorgo		F	Inflorescences peduncles	1	0	1	20		P
Polygonaceae	*Emex spinosa* (L.) Campd.	Acelguillas		F	Leaves	1	0	1	15		1
Polygonaceae	*Polygonum persicaria* L.	Hierba carbunquera, vinagrera		F	Leaves	2	0	2	6, 20		1; P
Polygonaceae	*Rumex acetosa* L.	Acedera, carnerillo, espinaca silvestre		F	Leaves	2	0	2	13, 21	FCT	1; 2; 3; P
Polygonaceae	*Rumex bucephalophorus* L.	Acecera, vinagrera, vinagretas		F	Leaves	5	0	5	6, 8, 14, 19, 20		1; P
Polygonaceae	*Rumex conglomeratus* Murray	Espinaca silvestre		F	Leaves	4	11	15	1, 3, 6, 15		1; 2; 3; P
Polygonaceae	*Rumex crispus* L.	Romanza, romanza vinagrera		F	Leaves	8	26	34	3, 5, 6, 7, 11, 15, 19, 20		1; 2; 3; P
Polygonaceae	*Rumex induratus* Boiss. and Reut.	Vinagrera		F	Leaves	2	8	10	1, 13		1; 3
				Sn	Leaves	1	1	2	1		
				D	Aerial parts	1	0	1	1		
Polygonaceae	*Rumex pulcher* L.	Alborraza, romanza, vinagrera de guitarra		F	Leaves	7	20	27	1,3, 8, 11, 14, 15, 16	FCT	1; 2; 3; P
Polygonaceae	*Rumex scutatus* L.	Acedera, vinagreras		F	Leaves	1	1	2	2		1; 3; P
Portulacaceae	*Portulaca oleracea* L.	Verdolaga		F	Leaves/Stems	13	37	50	1, 2, 3, 4, 5, 7, 11, 12, 14, 15, 16, 20, 21	FCT	1; 2; 3; P
Primulaceae	*Samolus valerandi* L.	Lechuguilla, pamplina		F	Leaves/Stems	1	0	1	15		1; 2; P
Raflesiaceae	*Cytinus hypocistis* (L.) L.	Melera		Sn	Flowers	1	0	1	ND		1; 3; P
Ranunculaceae	*Clematis vitalba* L.	Birgaza, bizarra		F	Tender shoots	1	0	1	20		1; 3; P
Ranunculaceae	*Ranunculus ficaria* L.	Botón de oro, hierba centella		F	Leaves	1	0	1	19		P
Rhamnaceae	*Rhamnus alaternus* L.	Durillo, aladierno		Se	Leaves/Stems	2	0	2	8, 9	Olives dressing	1
Rhamnaceae	*Ziziphus lotus* (L.) Lam.	Arto		F	Fruits	2	1	3	13, 19	FCT	1
Rosaceae	*Amelanchier ovalis* Medik.	Guillomo		F	Fruits	1	0	1	ND		1; 3; P
Rosaceae	*Cotoneaster granatensis* Boiss.	Durillo		F	Fruits	1	0	1	20		1; 3
Rosaceae	*Crataegus azarolus* L. +	Acerolo		F	Fruits	2	5	7	1, 20		P
Rosaceae	*Crataegus granatensis* Boiss.	Majoleto	Cold	F	Fruits	1	1	2	1		
Rosaceae	*Crataegus laciniata* Ucria	Majoleto		F	Fruits	1	0	1	ND		1; P
Rosaceae	*Crataegus monogyna* Jacq.	Majoleto, espino blanco	Cold; diarrhoea; appetizer	F	Fruits	13	23	36	1, 3, 5, 6, 7, 8, 9, 11, 14, 15, 16, 19, 20	FCT; NSA	1; 3; P
				F	Leaves	2	0	2	7, 11		
				D	Flowers	1	0	1	15		
Rosaceae	*Filipendula vulgaris* Moench.	Macuca, ánnica, filipéndula		F	Roots	1	0	1	2		P
Rosaceae	*Fragaria vesca* L.	Fresa		F	Fruits	1	0	1	20	Sparsely naturally distributed; cultivated	1; 3; P
Rosaceae	*Malus sylvestris* (L.) Mill.	Maguillo		F	Fruits	1	0	1	2		1; 3; P
Rosaceae	*Prunus avium* L.	Cerezo	Dysmenorrhoea	F	Fruits	5	5	10	1, 2, 9, 14, 20	EN	1; P
				Li	Fruits	2	4	6	1, 2		
Rosaceae	*Prunus domestica* L.+	Ciruelo		F	Fruits	4	1	5	6, 9, 14, 20		1; P
Rosaceae	*Prunus dulcis* (Mill.) D.A. Webb +	Almendro	Gastralgia; diarrhoea; constipation; hypercholesterolemia	F	Fruits	10	4	14	1, 2, 3, 4, 8, 9, 10, 13, 15, 20		P
				Sn	Flowers	3	0	3	3, 9, 14		
				Sn	Gum	2	0	2	1, 20		
Rosaceae	*Prunus insititia* L.	Endrino		F	Fruits	1	0	1	1	EN	1; 3; P
				Li	Fruits	2	1	3	1, 3		
Rosaceae	*Prunus mahaleb* L.	Cerecino		F	Fruits	1	0	1	2	EN	1; P
Rosaceae	*Prunus spinosa* L.	Endrino	Hyperglucemia	Li	Fruits	5	5	10	1, 2, 3, 9, 14	FCT	1; 3; P
				F	Fruits	5	5	10	1, 8, 9, 14, 20		
Rosaceae	*Prunus ramburii* Boiss.	Endrino		Li	Fruits	1	0	1	ND		
Rosaceae	*Pyrus bourgaeana* Decne	Piruétano		F	Fruits	5	9	14	5, 7, 11, 15, 19		1
				D	Flowers	1	1	2	11		
Rosaceae	*Rosa canina* L.	Tapaculo	Diarrhoea; cold; asthenia	F	Tender shoots	1	1	2	1		1; P
				Sn	Fruits	2	7	9	1, 20		
				Sn	Flowers	1	0	1	3		
Rosaceae	*Rosa pouzinii* Tratt.	Tapaculo	Kidney problems	Sn	Fruits	2	1	3	6, 8		1
				Sn	Flowers	1	0	1	3		
Rosaceae	*Rosa sempervirens* L.	Tapaculo		Sn	Fruits	2	0	2	9, 19		
Rosaceae	*Rubus ulmifolius* Schott	Zarzamora	Stomach or intestinal problems	F	Fruits	13	32	45	1, 2, 3, 5, 6, 7, 8, 11, 13, 15, 16, 19, 20	FCT	1; 3; P
				F	Tender shoots	5	5	10	1, 3, 8, 11, 16		
				Li	Fruits	5	1	6	1, 6, 7, 16, 20		
Rosaceae	*Rubus laciniatus* Willd.	Zarzamora	Stomach or intestinal problems*	F	Fruits	1	0	1	14		1; 2; P
				F	Tender shoots	1	0	1	14		
Rosaceae	*Sanguisorba minor* Scop. subsp. *magnolii* (Spach) Briq.	Fresillas, sanguisorba	Amenorrhea; stomach ache	F	Leaves	1	0	1	3		1; 2; P
Rosaceae	*Sanguisorba verrucosa* (G. Don) Ces.	Fresilla, solisorba	Amenorrhea; stomach ache	F	Leaves	1	0	1	8		1; 2
Rosaceae	*Sorbus aria* (L.) Crantz	Mostazo		F	Fruits	2	2	4	1, 14	EN	1; 3; P
Rosaceae	*Sorbus aucuparia* L.	Serval		F	Fruits	1	0	1	ND	EN	1; P
Rosaceae	*Sorbus domestica* L. +	Selvo	Diarrhoea	F	Fruits	3	5	8	1, 8, 14		1; P
				Li	Fruits	1	0	1	1		
Rubiaceae	*Rubia peregrina* L.	Zarzaparrilla real	Depurative; diuretic; eczema; circulatory problems	Li	Roots	1	0	1	3		
Ruscaceae	*Ruscus aculeatus* L.	Rusco brusco	Haemorrhoids	F	Fruits	1	0	1	19	NSA	1; 2; P
				F	Tender shoots	2	0	2	6, 19		
Salicaceae	*Populus nigra* L.	Chopo, chopo negro		Sn	Inflorescences	1	0	1	3		P
Scrophulariaceae	*Linaria latifolia* Desf.	Pan y quesillo		Sn	Flowers	1	0	1	1		
Solanaceae	*Lycium europaeum* L.	Cambrón		F	Tender shoots	1	0	1	14		1; P
Smilacaceae	*Smilax aspera* L.	Zarzaparrilla	Circulatory problems; gastralgia; pain	Li	Roots	1	5	6	1	FCT	1; P
				F	Tender shoots	1	0	1	15	NSA	
				D	Leaves/Roots	4	0	4	2, 6, 15, 20		
Taxaceae	*Taxus baccata* L.	Tejo		F	Fruits aril	1	0	1	ND	EN	1; P
Typhaceae	*Typha domingensis* Pers.	Anea, enea		F	Tender shoots	1	0	1	ND		P
Ulmaceae	*Celtis australis* L.	Almencino		F	Fruits	10	10	20	1, 2, 3, 5, 6, 7, 14, 17, 19, 20		1; 3; P
Ulmaceae	*Ulmus minor* Mill.	Álamo negro, olmo		Sn	Fruits	2	3	5	1, 14		1; 2; P
				F	Leaves	2	0	2	5, 7		
Urticaceae	*Urtica dioica* L.	Ortiga	Circulatory problems; diuretic	F	Aerial parts	4	4	8	1, 3, 6, 16	FCT	1; 2; 3; P
Urticaceae	*Urtica membranacea* Poir.	Ortiga		F	Aerial parts	4	0	4	3, 8, 18, 19		1
Urticaceae	*Urtica urens* L.	Ortiga	Circulatory problems; diuretic	F	Whole plant	5	4	9	1, 3, 5, 6, 7		1; 2; 3; P
Viscaceae	*Viscum album* L.	Muérdago, almuérdago	Hypertension	F	Fruits	2	0	2	2, 6		1; P
Vitaceae	*Vitis vinifera* L. subsp. *sylvestris* (C.C.Gmelin) Hegi	Parra silvestre, vid		F	Fruits	2	0	2	7, 11		1; 2; 3; P
				F	Tender shoots	1	0	1	11		
