# Supplementary material for: Wild Edible Plants of Andalusia: Traditional Uses and Potential of Eating Wild in a Highly Diverse Region
Source: Plants (Basel). 2023 Mar 7;12(6):1218. doi: 10.3390/plants12061218 (PMC10051205; doi:10.3390/plants12061218)
Supplement: Supplementary file 1 [file plants-12-01218-s001.zip › plants-2221778-supplementary.pdf]

Supplementary Table S1. Potential wild edible plants (PWEPs) of Andalusia.

Family	Scientific name	Vernacular name	Category	Part used	Ref.
Aizoaceae	*Aizoon hispanicum* L.	Agua azul, gazula	Food	Flowers	ND
Aizoaceae	*Carpobrotus acinaciformis* (L.) L. Bolus	Uña de gato	Food	Flowers, fruits	Ríos et al., 2021
Aizoaceae	*Carpobrotus edulis* (L.) N. E. Br.	Uña de gato	Food	Leaves, fruits and flowers	Ríos et al., 2021
Aizoaceae	*Mesembryanthemum crystallinum* L.	Aguazul, algazul, garullicos	Food	Flowers	Peris et al., 2019
Aizoaceae	*Mesembryanthemum nodiflorum* L.	Escarchada	Food	Flowers	Peris et al., 2019
Anacardiaceae	*Rhus coriaria* L.	Zumaque	Seasoning	Fruit	ND
Amaranthaceae	*Amaranthus blitum* L.	Bleo	Food	Leaves	Tardío et al., 2006
Amaryllidaceae	*Narcissus bulbocodium* L.	Narciso	Snack	Bulb	González et al., 2011
Apiaceae	*Ammi majus* L.		Food	Leaves	Hadjichambis et al., 2008
Apiaceae	*Anethum graveolens* L. +	Eneldo	Seasoning	Leaves	Peris et al., 2019
Apiaceae	*Carum carvi* L.	Alcaravea	Seasoning	Leaves	Tardío et al., 2006
Apiaceae	*Conopodium marianum* Lange	Chufera	Food	Tuber	Tardío et al., 2006
Apiaceae	*Coriandrum sativum* L. +	Cilantro	Food	Leaves	Tardío et al., 2006
Apiaceae	*Meum athamanticum* Jacq.	Perejil de monte	Seasoning	Leaves	Tardío et al., 2006
Apiaceae	*Pastinaca sativa subsp. sylvestris* (Mill.) Rouy & E. G. Camus	Chiviría	Food	Tuber	Peris et al., 2019
Apiaceae	*Physospermum cornubiense* (L.) DC.		Seasoning	Fruit	Pardo de Santayana et al., 2007
Asparagaceae	*Asparagus aphyllus* L.	Espárrago	Food	Tender shoots	Tardío et al., 2006
Asteraceaae	*Andryala ragusina* L.	Liria, liga	Snack	Latex	Tardío et al., 2002
Asteraceae	*Artemisia absinthium* L.	Ajenjo	Liqueur	Leaves & Flowers	Bonet & Vallès, 2002
Asteracaee	*Bellis annua* L.	Margarita	Food	Leaves & Tender shoots	Peris et al., 2019
Asteracaee	*Bellis perennis* L.	Margarita	Snack	Inflorescences	Menéndez Baceta et al., 21012
Asteraceae	*Carduus meonanthus* Hoffmanns. & Link	Cardo	Food	Leaves & Stems	Tardío et al., 2006
Asteraceae	*Cardus tenuiflorus* Curtis	Cardo	Food	Leaves & Stems	Tardío et al., 2002; Peris et al., 2019
Asteraceae	*Carthamus lanatus* L.	Cardo	Food	Leaves	Tardío et al., 2006; Oltra, 1999; Peris et al., 2019
Asteraceae	*Centaurea aspera* L.	Abrepuños, cardo, rompepiedras	Liqueur	Aerial part	Bonet & Vallès, 2002
			Drink	Aerial part	Bonet & Vallès, 2002
Asteraceae	*Centaurea calcitrapa* L.	Abrepuños	Food	Leaves	Tardío et al., 2006; Peris et al., 2019
Asteraceae	*Cirsium echinatum* (Desf.) DC.	Cardo	Seasoning	Inflorescences	Parada et al., 2011

Asteraceae	*Chamaemelum nobile* (L.) All.	Manzanilla romana	Drink, Liqueur	Inflorescence	Tardío et al., 2006; González et al., 2011
Asteraceae	*Chiliadenus glutinosus* (L.) Fourr.	Té de piedra	Drink	Flowered aerial parts	Tardío et al., 2006
Asteraceae	*Cynara humilis* L.	Alcacil	Food	Inflorescences	Rivera et al., 2006
Asteraceae	*Galactites tomentosa* Moench	Cardo, cardo de burro, cardo borriquero	Food	Leaves	Peris et al., 2019
Asteraceae	*Helminthotheca comosa* (Boiss.) Holub	Lenguaza	Food	Leaves	Tardío et al., 2006
Asteraceae	*Hypochaeris glabra* L.	Lechera, lechugueta	Snack	Stem	Tardío et al., 2002; Tardío et al., 2006
Asteraceae	*Lactuca muralis* (L.) Gaertn.	Lechuga silvestre	Food	Leaves	Peris et al., 2019
Asteraceae	*Lactuca virosa* L.	Lechuga silvestre	Food	Leaves	Peris et al., 2019
Asteraceae	*Lapsana communis* L.		Food	Leaves	Peris et al., 2019
Asteraceae	*Launaea nudicaulis* (L.) Hook. f	Rascamoño, papovieja, rascaviejas	Food	Leaves	Tardío et al., 2006; Peris et al., 2019
Asteraceae	*Leontodon saxatilis* Lam.	Almirón	Food	Leaves	Peris et al., 2019
Asteraceae	*Matricaria aurea* (Loefl.) Schultz Bip.	Manzanilla, Camomila	Liqueur	Flowered aerial parts	Díaz Fernández & del Monte, 2012
Asteracaee	*Notobasis syriaca* (L.) Cass.	Cardón	Food	Leaves & Tender shoots	Hadjichambis et al., 2008
Asteraceae	*Onopordum acanthium* L.	Cardo, toba	Food	Leaves midrib, stem	Rivera et al., 2006; Tardío et al., 2006
Asteraceae	*Onopordum corymbosum* Willk.	Cardo, toba	Food	Leaves midrib, stem	Rivera et al., 2006; Tardío et al., 2006
Asteraceae	*Reichardia intermedia* (SchultzBip.) Countnho	Cosconilla	Food	LeavesTender	Oltra, 1999; Tardío et al., 2006
Asteraceae	*Reichardia picroides* (L.) Roth	Cosconilla	Food	Tender leaves	Bonet & Vallès, 2002; Tardío et al., 2006; Peris et al., 2019
Asteraceae	*Reichardia tingitana* (L.) Roth	Cosconilla	Food	Tender Leaves	Oltra, 1999; Tardío et al., 2006
Asteraceae	*Rhagadiolus stellatus* (L.) Gaerner	Matamaridos	Food	Stem & Leaves	Rivera et al., 2006; Tardío et al., 2006
Asteraceae	*Sonchus aquatilis* Pouret	Borraja	Food	Tender shoots	Peris et al., 2019
Asteraceae	*Sonchus crassifolius* Pourret ex Willd.	Borraja	Food	Tender shoots	Tardío et al., 2002
Asteraceae	*Tanacetum balsamita L.*	Herba cuquera	Liqueur	Leaves	Bonet & Vallès, 2002
Asteraceae	*Tanacetum parthenium* (L.) Schultz	Manzanilla	Liqueur	Flowered aerial parts	Bonet & Vallès, 2002
Asteraceae	*Taraxacum dissectum* (Ledeb.) Ledeb.	Diente de león	Food	Leaves	Rigat et al., 2009
Asteraceae	*Tolpis barbata* (L.) Gaertn.	Almiones	Food	Leaves	Tardío et al., 2006
Asteraceae	*Tragopogon pratensis* L.	Tetica de vaca, lecherin	Food	Tender stems	Pardo de Santayana et al., 2005; Tardío et al., 2006

Boraginaceae	*Buglossoides arvensis* (L.) I. M. Johnston	Abremano	Food	Leaves	Tardío et al., 2006
Boraginaceae	*Echium plantagineum* L.	Viborera	Snack	Flowers	Tardío et al., 2006; González et al., 2011
Boraginaceae	*Echium vulgare* L.	Viborera	Snack	Flowers	Tardío et al., 2006; Gonzalez Barriuso, 2012; Peris et al., 2019
Boraginaceae	*Lithospermum officinale* L.	Mijo del sol	Snack	Flowers	Rivera et al., 2006
Brassicaceae	*Cakile maritima* Scop.		Food	Leaves	Peris et al., 2019
Brassicaceae	*Cardamine hirsuta* L.	Mastuerzo menor	Food	Leaves	Peris et al., 2019
Brassicaceae	*Crambe maritima* L.		Food	Leaves	Rivera and Obon, 1991
Brassicaceae	*Descurainia sophia* (L.) Webb ex Prantl	Ajenjo loco	Food	Leaves & tender shoots	Peris et al., 2019
Brassicaceae	*Diplotaxis catholica* DC.	Barelo	Snack	Flower	Tardío et al., 2002; Tardío et al., 2006
Brassicaceae	*Lepidium sativum* L. +	Mastuerzo	Food	Leaves	ND
Brassicaceae	*Raphanus raphanistrum* L.	Jaramago, rabano, rabanillo	Food	Leaves	Tardío et al., 2002; Tardío et al., 2006; Hadjichambis et al., 2008; Peris et al., 2019
Brassicaceae	*Sinapis arvensis* L.	Jaramago	Food	Leaves	Pardo de Santayana et al., 2007; Hadjichambis et al., 2008
Brassicaceae	*Sisymbrium crassifolium* Cav.	Tamarilla	Food	Leaves & tender shoots	Rivera et al., 2006; Tardío et al., 2006; Peris et al., 2019
Brassicaceae	*Sisymbrium erysmoides* Desf.	Tamarilla	Food	Leaves & tender shoots	Peris et al., 2019
Brassicaceae	*Sisymbrium orientale* L	Tamarilla	Food	Leaves & tender shoots	Peris et al., 2019
Cactaceae	*Opuntia dillenii* (Ker–Gawler) Haw.	Chumbera	Food	Fuits	ND
Campanulaceae	*Trachelium caeruleum* L.	Hierba de la viuda	Food	Leaves	Tardío et al., 2006; Peris et al., 2019
Cannabaceae	*Humulus lupulus* L.	Lúpulo	Food	Tender stems	Tardío et al., 2006; Hadjichambis et al., 2008
Caprifoliaceae	*Viburnum lantana* L.	Lantana, morrionera	Food	Fruit	Pardo de Santayana et al., 2005; Rivera et al., 2006; Tardío et al., 2006
Caryophyllaceae	*Herniaria glabra* L.	Arenaria	Liqueur	Aerial part	Tardío et al., 2006
Caryophyllaceae	*Silene secundiflora* Otth		Food	Leaves	Tardío et al., 2006
Caryophyllaceae	*Silene diversifolia* Otth		Food	Leaves	Tardío et al., 2006; Peris et al., 2019
Caryophyllaceae	*Silene latifolia* Poir.		Food	Leaves	Tardío et al., 2006
Chenopodiaceae	*Atriplex glauca* L.	Salao, salao blanco	Food	Leaves	Lagasca, 1817
Chenopodiaceae	*Atriplex halimus* L.	Armuelle	Food	Leaves	Lagasca, 1817
Chenopodiaceae	*Beta macrocarpa* Guss.	Acelgas	Food	Leaves	Bonet & Vallès, 2002

Chenopodiaceae	*Chenopodium ambrosioides* L.	Té	Liqueur	Leaves	Tardío et al., 2006
Chenopodiaceae	*Halimione portulacoides* (L.) Aellen	Barrilla fina	Food	Stems	Lagasca, 1817
Chenopodiaceae	*Halogeton sativus* (L.) Moq.	Barrilla fina	Food	Stems	Lagasca, 1817; Peris et al., 2019
Chenopodiaceae	*Microcnemum coralloides* (Loscos & Pardo) Font Quer		Food	Stems	ND
Chenopodiaceae	*Salicornia ramosissima* J. Woods	Alacranera	Food	Tender stems	Peris et al., 2019
Chenopodiaceae	*Salsola kali* L.	Barrilla, barrita, garranchuelo	Food	Tender stems	Ríos et al., 2021
Chenopodiaceae	*Salsola soda* L.	Barrilla	Food	Tender stems	Ríos et al., 2021
Chenopodiaceae	*Sarcocornia perennis* (Mill.) A. J. Scott subsp. *alpini* (Lag.) Castroviejo	Sosa, sapina	Food	Stems	ND
Chenopodiaceae	*Suaeda spicata* (Willd.) Moq.		Food	Stems	Rivera & Obón, 1991; Rivera et al., 2008
Cistaceae	*Cistus ladanifer* L.	Jara pringosa	Snack	Seeds	Tardío et al., 2006
Cistaceae	*Cistus monspeliensis* L.	Estepa negra	Seasoning	Aerial parts	Parada et al., 2011
Clusiaceae	*Hypericum perforatum* L.	Hierba de San Juan	Liqueur	Flowered aerial parts	Bonet & Vallès, 2002; Tardío et al., 2006
Colchicaceae	*Merendera montana* (L.) Lange	Quitameriendas	Food	Bulbs	Tardío et al., 2006
Convolvulaceae	*Convolvulus arvensis* L.	Correhuela	Food	Tender aerial parts	Rigat et al., 2009
Crassulaceae	*Sedum album* L.	Uña de gato	Snack	Leaves	Tardío et al., 2006
Crassulaceae	*Sedum sediforme* (Jacq.) Pau	Uña de gato	Food	Leaves	Tardío et al., 2006
Cupressaceae	*Juniperus communis* L.	Enebro	Liqueur	Galbule	Bonet & Vallès, 2002
			Seasoning	Galbule	Bonet & Vallès, 2002
Cucurbitaceae	*Bryonia dioica* Jacq.	Nueza, melón bravío, melonera, pepinillo del diablo	Food	Tender shoots	Tardío et al., 2002; Tardío et al., 2006; Rivera et al., 2006; Peris et al., 2019
Cynomoriaceae	*Cynomorium coccineum* L.	Cipote	Food	Inflorescence	Verde et al., 2017
Cyperaceae	*Schoenoplectus lacustris* (L.) Palla	Junco	Food	Leaves	Molina Fernández, 2003
Dipsacaceae	*Scabiosa atropurpurea* L.	Porilla, escabiosa	Liqueur	Flowered aerial parts	Bonet & Vallès, 2002
Ericaceae	*Arctostaphylos uva -ursi* (L.) Spreng. *	Gayuba	Food	Fruit	Pardo de Santayana et al., 2005; Tardío et al., 2006
Ericaceae	*Vaccinium uliginosum* L. var. *nana* Boiss.	Arándano	Food	Fuits	ND
Euphorbiaceae	*Euphorbia helioscopia* L.	Lecheinterna, lechetrezna	Curd	Latex	Tardío et al., 2006
Euphorbiaceae	*Euphorbia segetalis* L.	Lechetrezna	Curd	Latex	Tardío et al., 2006
Fabaceae	*Cytisus scoparius* (L.) Link	Escobón, retama negra	Liqueur	Flowers	Bonet & Vallès, 2002

Fabaceae	*Cytisus scoparius subsp. reverchonii* (Degen & Hervier) Rivas Goday & Rivas Mart.	Retamón, retama negra	Liqueur	Flowers	ND
Fabaceae	*Trifolium pratense* L.	Trebol	Snack	Flower	Tardío et al., 2002; Tardío et al., 2006
			Food	Leaves	
Fabaceae	*Trifolium repens* L.	Trebol	Snack	Flower	Tardío et al., 2002; Tardío et al., 2006
Fabaceae	*Pterospartum tridentatum* (L.) Willk.	Carquesa	Seasoning	Inflorescences	Tardío et al., 2006; Pardo de Santayana et al., 2007
Fabaceae	*Vicia lutea* L.	Alverja	Snack	Seeds	Tardío et al., 2002; Tardío et al., 2006
Fabaceae	*Vicia villosa* Roth.	Alverja de culebra	Snack	Seeds	Tardío et al., 2002
Fagaceae	*Quercus faginea* Lam.	Quejigo	Food	Fruit	Tardío et al., 2006
Fagaceae	*Quercus pyrenaica* Willd.	Roble	Food	Fruit	Tardío et al., 2006
Gentianaceae	*Centaurium erythraea* Rafn	Centaura	Liqueur	Aerial part	Bonet & Vallès, 2002; Tardío et al., 2006
Gentianaceae	*Gentiana lutea* L.	Genciana	Liqueur	Aerial part	Tardío et al., 2006
Hyacinthaceae	*Muscari comosum* (L.) Mill.	Nazareno	Food	Bulb	Hadjichambis et al., 2008
Hypolepidaceae	*Pteridium aquilinum* (L.) Kuhn	Helecho	Food	Tender stems	Menéndez Baceta et al., 21012
Iridaceae	*Romulea bulbocodium* (L.) Sebast. & Mauri	Curcubilla	Food	Bulb	Pardo de Santayana et al., 2005: Tardío et al., 2006
Lamiaceae	*Hyssopus officinalis* L.	Hisopo	Liqueur	Aerial part	Bonet & Vallès, 2002; Tardío et al., 2006
Lamiaceae	*Lamium amplexicalule* L.	Sombrillicas, zapaticos locos, pericón	Snack	Flower	Díaz Fernández & del Monte, 2013
Lamiaceae	*Lamium maculatum* L.	Mamatetas	Snack	Flower	Pardo de Santayana et al., 2005; Tardío et al., 2006
Lamiaceae	*Lavandula multifida* L.	Cantueso	Seasoning	Flowered aerial parts	Tardío et al., 2006
Lamiaceae	*Lavandula pedunculata* (Mill.) Cav.	Tomillo, tomillo de burro	Seasoning	Flowered aerial parts	Tardío et al., 2006; González et al., 2011
Lamiaceae	*Nepeta cataria* L.	Nepeta	Liqueur	Leaves	Bonet & Vallès, 2002
Lamiaceae	*Salvia argentea* L.	Gordolobo	Food	Tender shoots	Rivera et al., 2006; Tardío et al., 2006
Lamiaceae	*Salvia sclarea* L.	Salvia romana	Seasoning	Leaves & inflorescences	Pardo de Santayana et al., 2007
Liliaceae	*Lilium candidum* L. +	Azucena	Liqueur	Flower	Bonet & Vallès, 2002
Malvaceae	*Althaea officinalis* L.	Malvavisco	Liqueur	Root	Bonet & Vallès, 2002; Tardío et al., 2006
Malvaceae	*Lavatera maritima* Gouan	Malvavisco	Snack	Flowers and fruit	Peris et al., 2019

Malvaceae	*Lavatera arborea* Gouan	Malva arbórea, malvavisco	Snack	Flowers and fruit	Peris et al., 2019
Orobanchaceae	*Bartsia trixago* L.	Gallocresta	Snack	Flowers	Tardío et al., 2006
Oxalidaceae	*Oxalis acetosella* L.	Vinagrera	Food	Leaves	Tardío et al., 2006; Menéndez Baceta et al., 2012
Papaveraceae	*Papaver dubium* L.	Amapola	Food	Leaves	Peris et al., 2019
Papaveraceae	*Roemeria hybrida* (L.) DC.	Amapola morada	Food	Leaves	Rivera et al., 2006; Tardío et al., 2006
Poaceae	*Brachypodium retusum* (Pers.) Beauv.	Lastón	Food	Stems	Parada et al., 2011
Poaceae	*Phragmites australis* (Cav.) Trin. ex Steud.	Carrizo	Food	Rhizome	Ríos et al., 2021
Poaceae	*Celtica gigantea* (Link) F. M. Vázquez & Barkworth	Grama	Snack	Rhizome and Stem	Tardío et al., 2006
Plantaginacae	*Plantago coronopus* L.	Hierba de la piedra	Food	Leaves	Oltra, 1999; Tardío et al., 2006; Peris et al., 2019
Plantaginacae	*Plantago maritima* L.	Llantén	Food	Leaves	Ríos et al., 2021
Polygonaceae	*Fallopia convolvulus* L.	Albohol	Food	Tender shoots	Rivera et al., 2006; Tardío et al., 2006
Polygonaceae	*Rumex angiocarpus* Murb.	Acedera	Snack	Leaves and stem	Rivera et al., 2006
Polygonaceae	*Rumex intermedius DC.*	Vinagreras	Snack	Leaves and stem	Tardío et al., 2006; Peris et al., 2019
Polygonaceae	*Rumex obtusifolius L*	Paradelles, Santes Maries	Food	Aerial part	Bonet & Vallès, 2002; Tardío et al., 2006
Polygonaceae	*Rumex papillaris* Boiss. & Reuter	Acedera	Snack	Leaves	Tardío et al., 2006; Díaz Fernández & del Monte, 2013
Portulacaceae	*Montia fontana* L.	Pamplina, hierba de manantial	Food	Leaves	Tardío et al., 2002; Tardío et al., 2006; Bonet & Vallès, 2002
Primulaceae	*Primula elatior* (L.) L. subsp. *lofthousei* (Hesl.–Harr.) W.W.Sm. & Fletcher	Primavera	Snack	Flower	Pardo de Santayana et al., 2005; Tardío et al., 2006
Primulaceae	*Primula veris* L.	Primavera	Snack	Flower	Tardío et al., 2006; Peris et al., 2019
Ranunculaceae	*Clematis vitalba* L.	Birgaza, enredadera, jazmín bravío	Food	Tender shoots	Rivera et al., 2006
Rosaceae	*Rosa micrantha* Sm.	Rosal, escaramujo, tapaculos	Snack	Fruit	Tardío et al., 2006
Rosaceae	*Rosa pimpinellifolia* L.	Rosal, escaramujo, tapaculos	Snack	Fruit	Tardío et al., 2006
Rosaceae	*Rosa sicula* Tratt.	Rosal, escaramujo, tapaculos	Snack	Fruit	Tardío et al., 2006
Rosaceae	*Rubus caesius* L.	Zarza	Food	Fruit	Rivera et al., 2006; Tardío et al., 2006
Rosaceae	*Sorbus torminalis* (L.) Crantz	Mostajo	Snack	Fruit	Tardío et al., 2002
Rubiaceae	*Galium verum* L.	Cuajaleche	Curd	Aerial part	Tardío et al., 2006
Rubiaceae	*Dictamnus hispanicus* Willk.	Dictamo, taraguillo	Liqueur	Aerial part	Tardío et al., 2006

Rubiaceae	*Ruta angustifolia* Pers.	Rua, ruda	Liqueur	Aerial part	Tardío et al., 2006
Rubiaceae	*Ruta chalepensis* L.	Rua, ruda	Liqueur	Aerial part	Tardío et al., 2006
Scrophulariaceae	*Linaria hirta* (L.) Moench	Pan y queso	Snack	Flower	Rivera et al., 2006; Tardío et al., 2006
Solanaceae	*Solanum nigrum* L.	Tomatico	Food	Leaves	Hadjichambis et al., 2008
Tropaeolaceae	*Tropaeolum majus* L. +	Capuchina, campanicas	Food	Flower	Peris et al., 2019
Urticaceae	*Parietaria judaica* L.	Pelusilla, hirba de muro, albahaquilla	Food	Leaves	Peris et al., 2019
Valerianaceae	*Valerianella locusta* (L.) Laterr.	Canónigo	Food	Leaves	Díaz Fernández & del Monte, 2013; Peris et al., 2019
Veronicaceae	*Veronica anagallis-aquatica* L.	Berraza	Food	Stems	Molina Fernández, 2003; Tardío et al., 2006; Peris et al., 2019
Veronicaceae	*Veronica anagalloides* Guss.	Berraza	Food	Stems	Peris et al., 2019
Veronicaceae	*Veronica beccabunga* L.	Berraza, becabunga, berro macho	Food	Leaves	Rivera et al., 2006; Menéndez Baceta et al., 2012; Peris et al., 2019
Violaceae	*Viola odorata* L.	Violeta	Food	Flower	Tardío et al., 2006; Peris et al., 2019
